# Novel Buccal Xanthan Gum–Hyaluronic Acid Eutectogels with Dual Anti-Inflammatory and Antimicrobial Properties

**DOI:** 10.3390/gels11030208

**Published:** 2025-03-15

**Authors:** Valentina Anuța, Mihaela-Alexandra Nica, Răzvan-Mihai Prisada, Lăcrămioara Popa, Bruno Ștefan Velescu, Ioana Cristina Marinas, Diana-Madalina Gaboreanu, Mihaela Violeta Ghica, Florentina Iuliana Cocoș, Cristian Andi Nicolae, Cristina-Elena Dinu-Pîrvu

**Affiliations:** 1 Department of Physical and Colloidal Chemistry, Faculty of Pharmacy, “Carol Davila” University of Medicine and Pharmacy, 6 Traian Vuia Street, 020956 Bucharest, Romania; valentina.anuta@umfcd.ro (V.A.); mihaela.nica@drd.umfcd.ro (M.-A.N.); lacramioara.popa@umfcd.ro (L.P.); mihaela.ghica@umfcd.ro (M.V.G.); florentina.cocos@drd.umfcd.ro (F.I.C.); cristina.dinu@umfcd.ro (C.-E.D.-P.); 2 Innovative Therapeutic Structures Research and Development Centre (InnoTher), “Carol Davila” University of Medicine and Pharmacy, 6 Traian Vuia Street, 020956 Bucharest, Romania; bruno.velescu@umfcd.ro; 3 Department of Pharmacology and Clinical Pharmacy, Faculty of Pharmacy, “Carol Davila” University of Medicine and Pharmacy, 6 Traian Vuia Street, 020956 Bucharest, Romania; 4 Research Institute of the University of Bucharest—ICUB, University of Bucharest, 91–95 Spl. Independentei, 050095 Bucharest, Romania; ioana-cristina.marinas@icub.unibuc.ro (I.C.M.); gaboreanu.diana-madalina@s.bio.unibuc.ro (D.-M.G.); 5 Departament of Botany and Microbiology, Faculty of Biology, University of Bucharest, Splaiul Independentei 91-95, 050095 Bucharest, Romania; 6 National Institute for Research and Development in Chemistry and Petrochemistry—ICECHIM Bucharest, 202 Spl. Independentei, 060021 Bucharest, Romania; cristian.nicolae@icechim.ro

**Keywords:** eutectogels, Natural Deep Eutectic Solvent (NADES), buccal drug delivery, mucosal drug delivery, xanthan gum, hyaluronic acid, anti-inflammatory activity, antimicrobial activity

## Abstract

Buccal drug delivery systems often struggle with poor drug solubility, limited adhesion, and rapid clearance, leading to suboptimal therapeutic outcomes. To address these limitations, we developed a novel hybrid eutectogel composed of xanthan gum (XTG), hyaluronic acid (HA), and a Natural Deep Eutectic Solvent (NADES) system (choline chloride, sorbitol, and glycerol in 2:1:1 mole ratio), incorporating 2.5% ibuprofen (IBU) as a model drug. The formulation was optimized using a face-centered central composite design to enhance the rheological, textural, and drug release properties. The optimized eutectogels exhibited shear-thinning behavior (flow behavior index, *n* = 0.26 ± 0.01), high mucoadhesion (adhesiveness: 2.297 ± 0.142 N·s), and sustained drug release over 24 h, governed by Higuchi kinetics (release rate: 237.34 ± 13.61 μg/cm^2^/min^1/2^). The ex vivo residence time increased substantially with NADES incorporation, reaching up to 176.7 ± 23.1 min. An in vivo anti-inflammatory evaluation showed that the eutectogel reduced λ-carrageenan-induced paw edema within 1 h and that its efficacy was sustained in the kaolin model up to 24 h (*p* < 0.05), achieving comparable efficacy to a commercial 5% IBU gel, despite a lower drug concentration. Additionally, the eutectogel presented a minimum inhibitory concentration for Gram-positive bacteria of 25 mg/mL, and through direct contact, it reduced microbial viability by up to 100%. Its efficacy against *Bacillus cereus*, *Enterococcus faecium*, and *Klebsiella pneumoniae*, combined with its significant anti-inflammatory properties, positions the NADES-based eutectogel as a promising multifunctional platform for buccal drug delivery, particularly for inflammatory conditions complicated by bacterial infections.

## 1. Introduction

Buccal drug delivery holds significant promise for both localized and systemic therapies due to its ability to bypass the gastrointestinal tract and first-pass metabolism [1]. Nevertheless, current systems face challenges such as poor drug solubility, inadequate mucosal adhesion, and variable drug release, hindering optimal therapeutic outcomes [2,3]. These shortcomings reduce the therapeutic efficacy of buccal products [4] and highlight the need for advanced materials capable of sustaining drug release while maintaining the mechanical integrity in the oral environment [5,6].

In this work, we propose a hybrid eutectogel system composed of xanthan gum (XTG), hyaluronic acid (HA), and a sugar alcohol-based Natural Deep Eutectic Solvent (NADES) containing choline chloride, sorbitol, and glycerol (2:1:1 mole ratio). Ibuprofen (IBU), an anti-inflammatory and analgesic agent, serves as a model drug. By combining NADES’s ability to enhance drug solubility with the robust, mucoadhesive gel matrix formed by XTG and HA, we aim to improve the formulation’s rheological and mechanical properties while surmounting the typical limitations of buccal delivery, namely, the vulnerability of conventional gels to saliva-induced dilution and rapid drug washout.

NADESs have recently gained prominence for improving the solubility of poorly soluble drugs [7]. Composed of naturally occurring metabolites (e.g., sugars, organic acids, amino acids, choline derivatives) [8], NADESs have the capacity to form stable, liquid eutectic mixtures at ambient temperatures [9] and offer a safer, more environmentally friendly alternative to conventional organic solvents [10,11]. Their high solvent capacity, low toxicity, and biocompatibility make them ideal for pharmaceutical applications [12,13].

When incorporated into a gel matrix, they form eutectogels [14], hybrid systems that retain the advantages of both components [15], by combining the solubilization enhancement properties of NADESs with the structural and mechanical properties of gels [16]. This synergy of properties results in a new class of materials with significant potential across various fields [17].

From a pharmaceutical perspective, eutectogels combine the benefits of liquid eutectic systems, such as enhanced solubility and stability of active pharmaceutical ingredients (APIs) [18,19,20], with the mechanical stability, bioadhesion, and ease of application provided by the gel matrix [16,21]. Such features are particularly beneficial for mucosal delivery, where extended residence time and controlled release are central to therapeutic efficacy [22]. Moreover, because the buccal and oral mucosa are well-vascularized and bypass first-pass hepatic metabolism, eutectogels can facilitate both localized therapy and systemic drug delivery with greater bioavailability [22,23].

Recent work underscores the versatility of NADES-based eutectogels across pharmaceutical formulations. For instance, they have proven successful in topical delivery systems, where NADESs increase the permeation of anti-inflammatory drugs [24] and biomacromolecules [25] through the skin, leading to improved therapeutic outcomes. In transdermal patches, NADES-based eutectogels serve as both a solvent and adhesive matrix, enabling controlled release of drugs such as menthol and lidocaine across the skin barrier [26]. Additionally, NADES-based eutectogels are being explored for the oral mucosal delivery of vitamins and therapeutic peptides, where they improve drug stability, solubility, and absorption [27]. Emerging research also points to their potential in nasal [28] and ocular drug delivery [29], as well as in parenteral formulations [30], further expanding the scope of NADES eutectogels in drug delivery technologies. The mechanical properties of eutectogels, such as elasticity [31], strength, adhesiveness [32], and flexibility [33], can likewise be tailored to match the requirements of specific biomedical applications [34], making them suitable for tissue scaffolds, wound dressings, or drug delivery systems that must function reliably in situ [35].

Despite these promising avenues, the study of eutectogels presents several challenges [36]. Their synthesis is more complex than that of conventional semisolids, demanding precise control over the composition and rheological properties [37]. Questions about their long-term stability and degradation behavior also need thorough investigation. Moreover, scaling up production while ensuring product quality and uniformity represents a significant hurdle for broader commercialization [38].

Choosing the right polymeric components is essential for optimizing eutectogel performance. Xanthan gum (XTG) and hyaluronic acid (HA) are two biopolymers extensively studied and employed in pharmaceutical formulations due to their complementary and synergistic properties [39,40]. XTG, a high-molecular-weight polysaccharide derived from *Xanthomonas campestris* through microbial fermentation, is well known for its high viscosity, stability across a broad pH range, and shear-thinning behavior [41]. These properties make XTG an ideal candidate for creating a stable and spreadable gel matrix that can support and enhance the delivery of APIs.

Hyaluronic acid (HA), a naturally occurring glycosaminoglycan found in various connective tissues [42], is recognized for its remarkable water-retaining capacity, biocompatibility, and ability to promote tissue hydration, repair, and regeneration. HA’s properties make it particularly valuable in pharmaceutical formulations, where it enhances the bioadhesive characteristics of gels, ensuring the prolonged residence time and sustained release of drugs at the site of application [39,42]. When combined, XTG and HA form a robust gel network that not only supports the eutectic solvent system but also provides enhanced therapeutic potential through sustained release and strong adhesion to mucosal surfaces [43].

Given the potential of NADES-based eutectogels for mucosal drug delivery, this study aims to develop a novel hybrid eutectogel formulation to address buccal inflammatory conditions complicated by bacterial infections. The proposed system employs XTG and HA as gelling agents, a ternary NADES system (choline chloride/sorbitol/glycerol 2:1:1), and IBU as the active pharmaceutical ingredient. We employ a systematic optimization strategy focused on achieving desirable rheological, textural, and kinetic properties. Moreover, since oral inflammatory conditions are often exacerbated by microbial colonization [44], the dual advantage of this system lies in its potential antimicrobial as well as anti-inflammatory activity. We tested the hybrid eutectogels against Gram-positive (*Bacillus cereus*, *Enterococcus faecium*) and Gram-negative (*Klebsiella pneumoniae*) bacteria, chosen for their relevance to the oral cavity and for their distinct cell envelope structures, which can impede drug penetration [45]. By demonstrating inhibitory activity against representative strains from both groups, the eutectogels hold promise for broader therapeutic use where infection risk is high.

By addressing the need for buccal systems with enhanced mechanical integrity, antimicrobial efficacy, and sustained drug release, this research contributes to the growing field of next-generation mucosal delivery platforms, offering an advanced alternative for treating oral inflammatory conditions.

For clarity, all abbreviations used throughout this manuscript are detailed in the Supplementary Materials under the ‘List of abbreviations’.

## 2. Results and Discussion

### 2.1. Overview of Response Variables and Model Fitting

The effects of the water percentage (*X*_1_), HA percentage (*X*_2_), and XTG percentage (*X*_3_) on the rheological, textural, and drug release properties of hybrid XTG-HA eutectogels were analyzed using a face-centered central composite design. This experimental design enabled the exploration of the linear, quadratic, and interaction effects of the independent variables on the 12 response variables (*Y*_1_ to *Y*_12_). The experimental results for all 12 response variables included in this study, expressed as the average ± SD are presented in Table 1. The results were modeled using a second-order polynomial equation, with non-significant terms iteratively removed through backward elimination based on the Akaike information criterion (AIC).

The multiple regression analysis yielded models with high significance (*p* < 0.0001) for all response variables, indicating that the polynomial models adequately captured the relationships between the independent variables and the measured responses (Table 2). The lack of fit was non-significant for all models, confirming that the models fit the experimental data well. The adjusted *R*-squared values were high (between 0.9096 and 0.9886), demonstrating that the models explained a substantial proportion of the variability in the response variables (Table 2).

The signs and magnitudes of the regression coefficients provide insights into the effects of the independent variables on the response variables. A positive coefficient indicates that an increase in the corresponding independent variable leads to an increase in the response variable. A negative coefficient indicates that an increase in the corresponding independent variable leads to a decrease in the response variable.

### 2.2. Rheological Characterization

#### 2.2.1. Viscosity and Flow Properties

**Figure 1:**
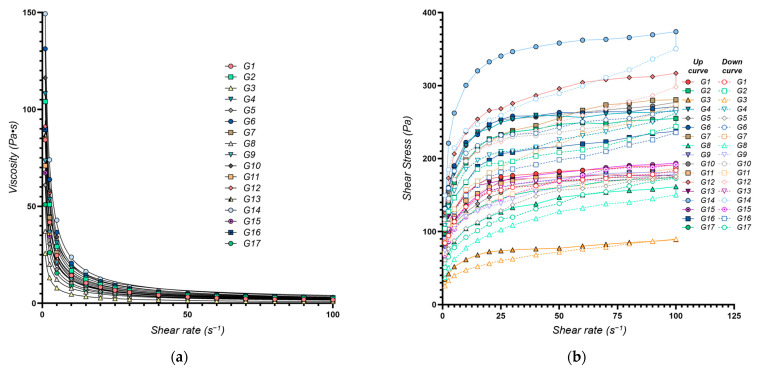
Rheological properties of the XTG-HA eutectogels: (**a**) Viscosity as a function of shear rate. The continuous lines represent the power law fittings for each formulation, highlighting the shear-thinning behavior typical of these gels; (**b**) Shear stress as a function of shear rate. Both the upward (solid symbols) and downward (open symbols) curves are plotted, illustrating the hysteresis loop observed in each formulation.

The flow behavior of the eutectogels was characterized using the power law model, yielding the consistency index (*K*, *Y*_1_) and the flow behavior index (*n*, *Y*_2_). A high degree of model fit was achieved (*R*^2^ > 0.99 for all formulations) (Figure 1a). The calculated values for both *K* and *n* are presented in Table 1.

The consistency index (*K, Y*_1_), an indicator of gel viscosity at low shear rates, ranged from 25.8 Pa·s^n^ (G3) to 150.4 Pa·s^n^ (G14), reflecting a broad spectrum of flow behaviors. Higher *K* values (e.g., G14 with 150.4 Pa·s^n^) signify a thicker, more viscous, structured gel, whereas lower values (e.g., G3 with 25.8 Pa·s^n^), correspond to a more fluid system. The multiple regression model (adjusted *R*^2^ value of 0.9761) confirmed that the chosen factors explain most of the observed variability in *K*. These findings align with prior studies on polysaccharide-based gels, which have reported comparable viscosity ranges and underscored the importance of the formulation components in shaping the rheological properties, drug release profiles, and therapeutic efficacy [46,47].

The water content (*X*_1_) exerted a negative effect on the *K* values (*β* = −8.952, *p* < 0.0001), indicating that increasing the percentage of water in the formulation decreased the consistency index. This is consistent with water’s “plasticizing” role: by increasing intermolecular spacing, it reduces chain entanglement and thus lowers viscosity [48]. This finding aligns with the existing literature, which highlights the role of water in disrupting intermolecular interactions within polymer matrices, leading to a more fluid-like system. For example, Xia et al. demonstrated that water is critical in the formation of eutectogels, influencing both their viscoelastic properties and shear-thinning behavior [36]. Their study highlighted that the addition of water improved the rheological characteristics of XTG-based eutectogels, resulting in enhanced thermal stability and defined shear-thinning behavior [36]. This supports the observation that an increased water content reduces both the *K* and *n* values, suggesting a transition towards a more Newtonian fluid behavior.

Conversely, HA incorporation (*X*_2_) had a significant positive effect on *K* (*β* = 24.167, *p* < 0.0001), which can be attributed to HA’s high molecular weight and strong hydrophilicity, which enable it to form a highly entangled network within the eutectogel [49]. For instance, Parsana et al. reported that the mechanical behavior and strength of supramolecular eutectogels are heavily influenced by HA, reinforcing the assertion that HA significantly contributes to the consistency index and the overall gel structure [50].

However, while previous studies have often focused on the linear impact of HA on rheological properties, our results suggest a more nuanced relationship. Specifically, HA also exhibited a significant positive quadratic effect (*β* = 6.528, *p* = 0.020), indicating that the increase in viscosity becomes more pronounced at higher HA concentrations. This non-linear relationship suggests that higher levels of HA lead to a more stabilized and reinforced network structure, further enhancing the gel’s resistance to flow. This phenomenon is consistent with the previous literature, which has reported that the entanglement of HA chains contributes to the viscoelastic properties of gels, leading to improved mechanical performance [51,52]. The entanglements are reported to act as physical crosslinks that can dissipate energy and accommodate large deformations without permanent damage, thus also improving the mechanical resilience of the gels [51,53].

XTG (*X*_3_) played a key role as the primary structuring agent in the eutectogels, owing to its ability to form helical structures and intermolecular entanglements [54]. Composed of repeating units of glucose, mannose, and glucuronic acid, XTG creates a robust, rod-like molecular network through a combination of hydrogen bonding and hydrophobic interactions [55]. This molecular architecture contributes to the formation of a stable, high-viscosity gel network, particularly at low shear rates [54]. Our findings showed that increasing XTG concentrations (*X*_3_) resulted in an increased consistency index (Table 1), which indicates that the system becomes more viscous and structured with greater amounts of XTG. This aligns with the well-documented thickening and gelling properties of XTG in solution [56,57]. Our results are also consistent with the findings of Xia et al., who reported that the viscoelastic properties of XTG solutions are influenced by its concentration, with higher concentrations resulting in greater elasticity and toughness [36].

The flow behavior index (*n*) reflects how markedly the gel shear-thins, with lower *n* values denoting stronger shear-thinning. Across the 17 formulations, *n* ranged from 0.186 ± 0.005 to 0.319 ± 0.018 (Table 1), indicating varying degrees of shear-thinning behavior across the different formulations, which is essential for applications requiring easy spreadability, such as topical gels [43]. Formulations with low *n* values (e.g., G13, *n* = 0.186) exhibit a significant viscosity reduction under shear, making them ideal for applications requiring easy spreadability, such as topical gels. Conversely, higher *n* values (e.g., G12, *n* = 0.319) indicate greater viscosity retention under shear, beneficial for products demanding structural integrity at rest. The multiple regression model adequately captured the variability in the flow behavior index (adjusted *R*^2^ = 0.9337).

Similar to its effect on *K*, water (*X*_1_) showed a significant negative effect on *n* (*β* = −0.020, *p* < 0.0001), consistent with a tendency toward more Newtonian flow at high water levels. This can be explained by the reduced interaction and entanglement of polymer chains in the presence of excess water, leading to a more fluid-like response under shear [36]. The positive quadratic effect of water on *n* further suggests that this trend becomes more pronounced at higher water concentrations.

The negative coefficient associated with XTG in the multiple regression model (*β* = −0.042, *p* < 0.0001) indicates its significant role in enhancing shear-thinning behavior, as it allows the gel to align under shear, thereby reducing viscosity [58]. This behavior is also consistent with the findings of Wang et al., who highlighted the importance of the conformational rigidity of polysaccharides such as XTG in determining the rheological properties of the resulting hydrogels [59].

The interaction between XTG and water also plays a crucial role in modulating the *n* values (*β* = 0.011, *p* = 0.013), with an increased water content leading to a more Newtonian behavior, primarily due to reduced polymer chain entanglement and the dilution effect of water [36,43]. This observation is also supported by the work of Zheng et al., who noted that the interaction between XTG and water can significantly influence the rheological properties of various semisolid systems [60].

The lack of a significant effect of HA on *n* can be attributed to the dominant role of XTG in dictating the shear-thinning behavior, as HA primarily enhances viscosity and adhesion rather than influencing shear-thinning [58,61,62].

Overall, water, HA, and XTG exhibit a complex interplay in modulating both viscosity and shear-thinning behavior. Acting primarily as a plasticizer [63], water reduces *K*, whereas HA and XTG each increase *K*, albeit in slightly different ways: HA’s high molecular weight and hydrophilicity promote chain entanglement [49], and XTG’s rigid backbone adds structural reinforcement at low shear rates [64,65]. This synergy aligns with findings on polysaccharide-based or dual-polymer gels, where complementary polymers form more stable, entangled networks [41,66].

Regarding flow behavior, our data showed that XTG strongly drives shear-thinning (*n* < 1), consistent with prior findings where polysaccharides such as xanthan or guar gum exhibit greater shear-thinning due to the alignment of polymer chains under shear [58,59]. HA did not markedly impact *n* in our design space, presumably due to XTG’s dominant conformational rigidity overshadowing HA’s contribution to shear-thinning [43,62]. These observations underscore the importance of polymer selection when targeting specific flow profiles in mucosal or topical preparations.

The shear-thinning behavior of the eutectogel plays a crucial role under physiological conditions, ensuring optimal performance in buccal drug delivery. At rest, the gel maintains high viscosity, preventing premature drug leakage [36]. However, upon exposure to shear forces from tongue movements, swallowing, and saliva flow, it becomes less viscous, facilitating easy application and uniform spreading. Once applied, the gel rapidly recovers its structure, maintaining prolonged adhesion and sustained drug release despite the dynamic intraoral environment [67].

The presence of significant interaction terms in the regression models highlights the complex interplay between these components, underscoring the importance of considering their combined effects when tailoring the rheological properties of these eutectogels [68].

#### 2.2.2. Thixotropy Evaluation

Thixotropy is a time-dependent shear-thinning property, where a material becomes less viscous when subjected to shear stress and gradually recovers its viscosity when the stress is removed [69]. This property is especially desirable in pharmaceutical semisolids, as it allows for easy spreading during application while retaining structural integrity at rest [70]. In this study, we evaluated the thixotropic properties of XTG–HA eutectogels through the hysteresis loop area (*S_thix_*, *Y*_3_) and the thixotropy index (*TI*, *Y*_4_). Together, these parameters offer insights into the structural breakdown under shear and the reversibility of that breakdown once shear is removed [70].

*S_thix_* quantifies the energy loss due to internal structural breakdown and recovery during a complete cycle of increasing and decreasing shear rates [71]. It is a direct measure of the material’s thixotropy, where a larger hysteresis loop area indicates greater thixotropic behavior [72].

*TI* is a parameter that quantifies the extent of viscosity recovery after shear has been applied, expressing how effectively the gel restores its viscosity post-shear [73]. It is calculated by normalizing *S_thix_* by the total area under the upward curve in a shear rate vs. shear stress plot, thus providing a dimensionless, relative measure of thixotropy, enabling comparisons across different formulations [69].

By combining these two measures, we obtain a more robust and nuanced understanding of the thixotropic properties of the NADES eutectogels, capturing both the absolute and relative aspects of their behavior under shear [70].

**Figure 2:**
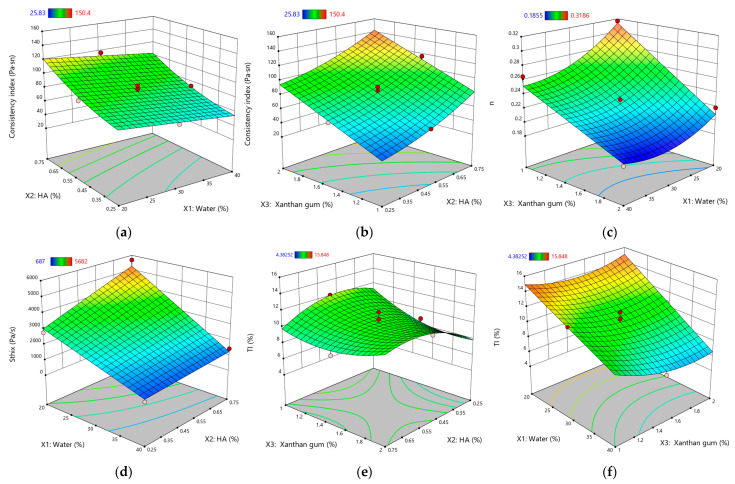
Response surface plots for rheological and thixotropic properties of XTG-HA eutectogels: (**a**) Consistency index (Pa·sⁿ) as a function of water (%) and HA (%); (**b**) Consistency index (Pa·sⁿ) as a function of XTG (%) and HA (%); (**c**) Flow behavior index (*n*) as a function of water (%) and XTG (%); (**d**) *S_thix_* (Pa·s^−1^) as a function of water (%) and HA (%); (**e**) TI (%) as a function of XTG (%) and HA (%); (**f**) TI (%) as a function of water (%) and XTG (%). Only graphs including very significant influences (*p* < 0.0001) are depicted.

Figure 2 illustrates the response surface plots for the rheological and thixotropic properties of XTG-HA eutectogels, highlighting only the most statistically significant effects observed in this study.

The measured *S_thix_* values ranged from 687 ± 51 Pa·s⁻^1^ (G3) to 5682 ± 105 Pa·s⁻^1^ (G14), indicating a broad spectrum of thixotropic responses across formulations. A higher value, as seen for G14, signals greater energy dissipation during shear and a slower return to baseline viscosity, reflecting robust structural networks that are more resistant to irreversible breakdown. Low *S_thix_*, (e.g., G3), corresponds to weaker thixotropy, where the gel’s internal structure is easily disrupted but also recovers quickly.

As anticipated, increasing the water content (*X*_1_) significantly reduced *S_thix_*. The dilutive effect of water weakens intermolecular interactions, minimizing the energy required for network disruption. This is consistent with the findings of Xia et al., who noted that water dilution in polysaccharide-based gels dampens viscoelastic strength and subsequently lowers overall thixotropy [36]. Furthermore, previous research has demonstrated that excess water can facilitate a transition from gel-like to more liquid-like behavior, which adversely impacts the gel’s structural integrity and its ability to recover from shear stress [52,74].

Conversely, both HA (*X*_2_) and XTG (*X*_3_) significantly enhance the hysteresis loop area, signifying greater thixotropy. HA’s intrinsic properties, including its high molecular weight and strong hydrophilic character, promote robust chain entanglement, contributing to a resilient gel network that resists disruption under shear forces [42]. This behavior aligns with prior studies that have shown HA’s potential to improve the mechanical and rheological properties of polysaccharide gels [75]. Similarly, XTG, known for its capability to form stable and elastic networks, requires greater energy for structural disruption and recovery, thus further enhancing the thixotropic response within the eutectogel [76].

Notably, we observe significant interaction effects. The negative interaction between water and both HA (*X*_1_*X*_2_) and XTG (*X*_1_*X*_3_) suggests a balancing act: while HA and XTG promote thixotropy, the simultaneous increase in the water content partially counteracts this effect through network dilution. These findings are in line with the observations of Sorze et al., who discuss the interplay between the water content and thixotropic properties in composite systems [77]. Furthermore, the positive quadratic effect of XTG ($X_{3}^{2}$) highlights its potent influence on thixotropy, with higher concentrations leading to a disproportionately larger hysteresis loop area. This aligns with the existing literature emphasizing XTG’s crucial role in the structural and rheological properties of gels [23,78,79].

*TI* exhibited values ranging from 4.38 ± 0.08% (G13) to 15.85 ± 1.02% (G14), reflecting substantial variability in the structural regeneration capacity of these eutectogels. Notably, G14 exhibited simultaneously high *S_thix_* and *TI*, indicating a well-balanced interplay of breakdown and recovery: the gel’s robust matrix demands significant energy to disrupt—primarily owing to the elevated HA and XTG concentrations—yet it still demonstrates efficient post-shear rebound. Such a combination of high energy dissipation and swift re-entanglement is often sought in pharmaceutical semisolids, as it ensures easy application without compromising longer-term mechanical stability [70].

Conversely, increasing the water content (*X*_1_) significantly reduced TI, underscoring how network dilution hampers the gel’s ability to regain its initial viscosity. While HA (*X*_2_) demonstrates a positive effect on the TI value, this effect is not statistically significant. This suggests that HA’s contribution to structural recovery, while present, is less pronounced than its impact on overall viscosity.

By contrast, XTG (*X*_3_) displayed a negative main effect on *TI*, which may seem counterintuitive given its strong viscosity-enhancing qualities and contribution to high *S_thix_*. However, this outcome aligns with the slow relaxation kinetics documented for rigid polysaccharides, where lengthy polymer chains require additional time to reform after shear disruption [80,81].

Examining the interaction effects provides further insights. The negative interactions between water and both HA (*X*_1_*X*_2_) and XTG (*X*_1_*X*_3_) suggest that the dilutive effect of water can partially offset the structural enhancements provided by these polymers, leading to a reduced thixotropy index. In contrast, the positive interaction between HA and XTG (*X*_2_*X*_3_) underscores a synergistic effect, where their combined presence promotes a more substantial recovery of viscosity. This synergy is echoed in studies on biopolymer blends—particularly those involving multiple polysaccharides—where complementary polymer networks often outperform single-polymer matrices in terms of mechanical resilience and recovery [58,77].

Meanwhile, the negative quadratic effect of water ($X_{1}^{2}$) illustrates that excessive dilution more sharply undermines re-entanglement, aligning with prior reports highlighting how free water disrupts inter-polymer junctions, diminishes the energy dissipation capacity, and hinders immediate viscosity recovery [36,77,82].

By contrast, the positive quadratic effect of XTG ($X_{3}^{2}$) indicates that sufficiently high XTG levels can significantly boost the network’s regenerative potential once shearing ceases—an effect reported previously in rigid polysaccharide systems where rod-like chains, at or beyond a threshold concentration, solidify the gel’s microstructure [23,40].

Taken together, water, HA, and XTG each exert a multifaceted impact on the thixotropic behavior of NADES-based gels. Water typically impairs structural regeneration via dilution, whereas HA and XTG strengthen both the gel matrix and its thixotropic potential through entanglement. Crucially, these effects do not act in isolation: interactions among the components can amplify or dampen individual contributions. Similar findings have been noted in composite hydrogel research, where balancing polymer concentrations and hydration levels proved essential to achieving the desired compromise between shear resistance and rapid recovery [17,70,83]. Therefore, formulation optimization should carefully consider both linear and quadratic effects, particularly if a gel requires robust shear resistance alongside rapid viscosity recovery in practical applications.

Consequently, formulation optimization must carefully consider both linear and non-linear relationships, particularly if a gel demands robust shear resistance while still allowing prompt viscosity recovery post-shear. This dual requirement is critical in many pharmaceutical and biomedical applications—ranging from mucosal drug delivery to wound dressings—where materials must apply smoothly and yet maintain stable mechanical and functional properties in situ [70].

### 2.3. Texture Analysis

The textural properties of XTG-HA eutectogels are critical for their performance in pharmaceutical and cosmetic applications. These properties, including the hardness (*Y*_5_), adhesiveness (*Y*_6_), cohesiveness (*Y*_7_), resilience (*Y*_8_), springiness (*Y*_9_), and stringiness (*Y*_10_), provide insight into the mechanical behavior and user experience of the gels [52,84]. These mechanical attributes not only affect user experience—for instance, how easily a gel can be applied or removed—but also influence drug release, mucosal retention, and overall stability [85,86,87].

**Figure 3:**
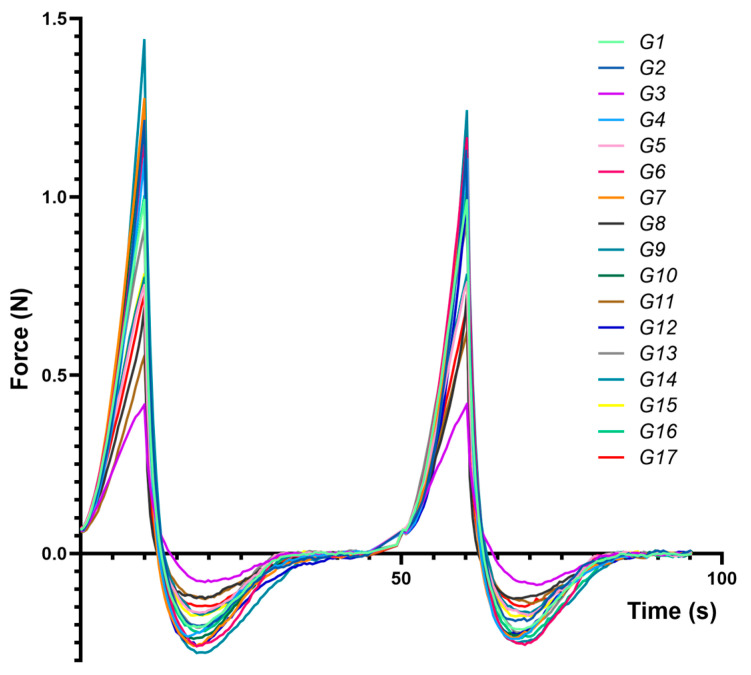
Force vs. time average texture analysis traces obtained for the experimental eutectogels during two compression–decompression cycles, using a cylindrical 25 mm probe.

Figure 3 presents compression–decompression profiles for each formulation (G1–G17), highlighting distinct responses to mechanical stress and underscoring differences in hardness, adhesiveness, and cohesiveness.

Table 2 summarizes the significant regression terms, detailing how the independent variables (water content, HA, XTG) shape the textural outcomes.

**Figure 4:**
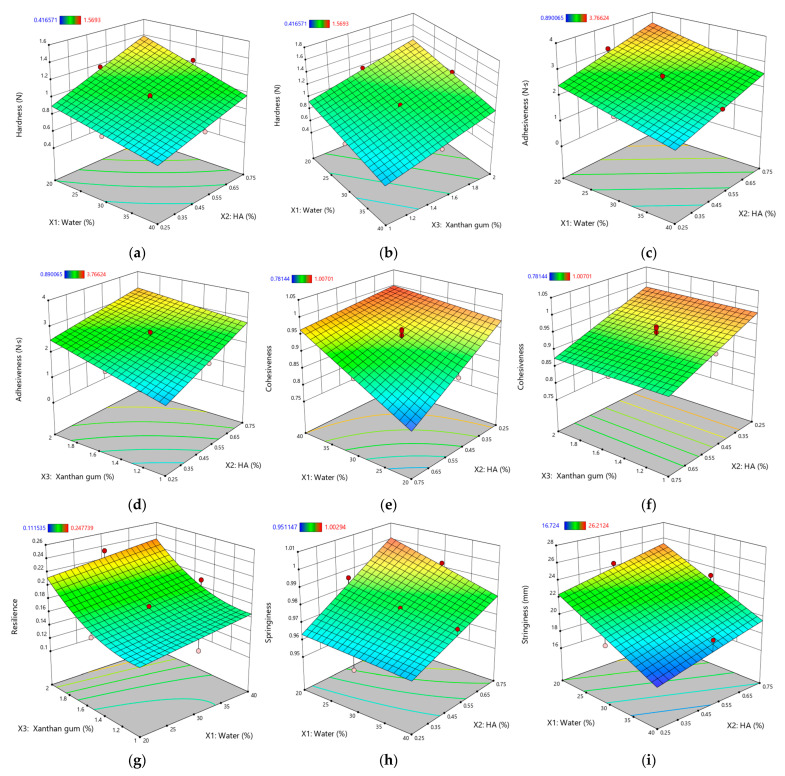
Response surface plots for textural properties of XTG-HA eutectogels: (**a**) Hardness (N) as a function of water (%) and HA (%); (**b**) Hardness (N) as a function of water (%) and XTG (%); (**c**) Adhesiveness (N·s) as a function of water (%) and HA (%); (**d**) Adhesiveness (N·s) as a function of water (%) and XTG (%); (**e**) Cohesiveness as a function of water (%) and HA (%); (**f**) Cohesiveness as a function of XTG (%) and HA (%); (**g**) Resilience as a function of water (%) and XTG (%); (**h**) Springiness as a function of water (%) and HA (%); (**i**) Stringiness (mm) as a function of water (%) and HA (%). Only graphs including very significant influences (*p* < 0.0001) are depicted.

To further clarify these influences, response surface plots (Figure 4) were generated for parameters with very high significance (*p* < 0.0001). This focused approach avoids an overabundance of graphics yet provides insight into the most critical interactions.

#### 2.3.1. Hardness (*Y*_5_)

Gel hardness (*Y*_5_), reflecting the structural integrity and resistance to deformation [52], ranged from 0.689 ± 0.044 N (G8) to 1.569 ± 0.084 N (G14) and was profoundly influenced by the eutectogel composition. As expected, water (*X*_1_) had a significant negative effect (*p* < 0.0001), consistent with its dilutive impact on the polymer network, mirroring similar trends noted in the consistency index (*Y*_1_) analysis.

In contrast, both HA (*X*_2_) and XTG (*X*_3_) boosted hardness, with G14 attaining the highest value. This highlights their role in strengthening the gel matrix through network formation. Notably, a synergistic interaction between HA and XTG was observed, further amplifying their individual contributions to gel strength. This synergy likely arises from the complementary nature of their molecular architectures, with HA’s long chains and hydrogen bonding capacity intertwining with XTG’s helical structure to create a robust and interconnected network [54,88].

Interestingly, a negative interaction (*X*_1_*X*_2_) between water and HA was observed, indicating that excessive water counteracts HA’s thickening role and underscoring the importance of balancing water and HA for optimal hardness. Similar observations have been reported in composite polysaccharide formulations, where a precise water content ensures polymer–polymer interactions remain favorable for maintaining structural integrity [36].

#### 2.3.2. Adhesiveness (*Y*_6_)

The adhesiveness (*Y*_6_), quantifying how strongly a gel interacts with surfaces [89], varied from 0.89 ± 0.07 N·s (G3) to 3.49 ± 0.26 N·s (G12). Similar to hardness, water (*X*_1_) negatively impacted adhesiveness (*p* < 0.0001), reinforcing the idea that a less viscous gel exhibits weaker interfacial interactions [90]. Both HA (*X*_2_) and XTG (*X*_3_) increased adhesiveness (*p* < 0.0001 for both), likely through cohesive polymer networks that cling to surfaces. Such gel–surface adhesion enhancements are frequently observed in polysaccharide-based gels, where entangled networks facilitate intimate contact with application sites [52].

However, the negative interaction between HA and XTG (*p* = 0.005) implies possible competition for binding sites at elevated concentrations or localized network alterations, highlighting the nuanced relationships among these polymers.

#### 2.3.3. Cohesiveness (*Y*_7_)

The cohesiveness (*Y*_7_), a measure of the gel’s internal bonding strength [64], ranged from 0.781 ± 0.050 (G12) to 1.007 ± 0.102 (G3). Interestingly, water (*X*_1_) demonstrated a positive effect on cohesiveness (e.g., G3), potentially due to optimized hydration facilitating network interactions. This counterintuitive observation suggests that balanced hydration is crucial for maintaining gel structure, potentially by facilitating optimal polymer chain interactions [91].

Conversely, HA (*X*_2_), despite enhancing viscosity and hardness, negatively impacted cohesiveness (*p* < 0.0001). This finding implies that HA, at higher concentrations, might disrupt the uniformity of the gel matrix, potentially due to the formation of large aggregates or uneven distribution within the network. Similar patterns have been noted in other HA-based blends, where disproportionate HA levels can yield local inhomogeneities and reduced internal bonding [92,93]. Further, the specific crosslinking technology employed can alter HA gel cohesiveness: formulations produced with cohesive polydensified matrix (CPM) technology generally exhibit greater cohesiveness, whereas non-animal-stabilized HA gels are often less cohesive [92]. Additionally, the HA molecular weight and concentration each play pivotal roles [94], with higher HA concentrations frequently leading to decreased cohesiveness—likely owing to polymer chain overcrowding or aggregate formation that hampers uniform network formation [95]. These observations are consistent with our findings, where excessive HA disrupted the gel matrix and reduced cohesiveness in the XTG-based eutectogels.

A positive interaction between water and HA (*p* < 0.0001) was also observed, indicating that, at certain ratios, water and hyaluronic acid (HA) can collectively optimize cohesion, presumably by mediating polymer–polymer interactions and preventing excessive aggregation, thus creating a more homogeneous network structure. This phenomenon aligns with a broader body of literature underscoring HA’s strong affinity for water and its profound influence on hydration dynamics.

Thus, HA is often described as a structure maker, effectively organizing water molecules and enhancing their thermal stability [96]. Variations in the HA concentration alter the distribution of water species (characterized by distinct hydrogen-bonding environments), underscoring HA’s notable capacity to structure water [96,97]. Studies reveal that HA organizes water molecules into extended, ordered hydration shells—reaching up to 475 nm—and slows water reorientation, thus increasing overall viscoelasticity [98].

Notably, HA interacts more strongly with water than with its own chains, favoring a water-over-itself preference, which helps avert polymer self-aggregation [99,100]. Such HA–water synergy proves especially valuable in pharmaceutical and biomedical contexts, where robust yet flexible gels are needed for consistent drug delivery or wound protection [101]. Modulating water levels and HA ensures cohesive networks that maintain structural integrity under stress while also exhibiting good handling and viscoelastic properties [100]. Consequently, tuning this delicate balance can markedly influence gel performance, patient comfort, and therapeutic efficacy.

#### 2.3.4. Resilience (*Y*_8_)

The resilience (*Y*_8_), which quantifies the gel’s ability to recover its shape after deformation, ranged from 0.112 ± 0.007 (G8) to 0.248 ± 0.018 (G14). All three components—water (*X*_1_), HA (*X*_2_), and XTG (*X*_3_)—positively influenced resilience, with XTG’s effect being the most significant (*p* < 0.0001). This trend highlights the role of these components in reinforcing the gel network, ensuring that the material retains its integrity after mechanical stress. These findings are consistent with studies on biopolymer-based hydrogels, where structural recovery is attributed to intermolecular interactions and entanglement density [102,103].

The negative interaction (*X*_1_*X*_2_) between water and HA at higher concentrations suggests that excessive hydration may lead to over-stabilization of the gel network, thereby reducing its ability to recover after deformation. This effect aligns with reports on HA-based gels, where excessive water disrupts optimal crosslinking, leading to a less elastic structure [104,105]. Over-hydration may result in weaker interchain interactions, which could explain the observed diminished resilience in formulations with high water-to-HA ratios [104,106].

Interestingly, the positive quadratic effect ($X_{3}^{2}$) of XTG suggests that beyond a threshold concentration, XTG exponentially enhances resilience. This could be due to the formation of a highly entangled, elastic network, which allows the gel to better absorb stress and return to its original state. Similar trends have been reported for XTG-based hydrogels, where higher XTG concentrations promote intermolecular entanglements, reinforcing the gel matrix and improving elastic recovery, thus enhancing their mechanical stability and self-recovery [104,106].

#### 2.3.5. Springiness (*Y*_9_)

The springiness (*Y*_9_), which quantifies the extent of shape recovery after deformation, ranged from 0.951 ± 0.053 (G13) to 1.003 ± 0.037 (G12), indicating good elastic recovery across all formulations. Water (*X*_1_) and XTG (*X*_3_) negatively impacted springiness, by creating a denser and more rigid matrix. This is due to enhanced intermolecular interactions and network structuring, which limit the gel’s ability to recover its shape after deformation [107]. These findings are consistent across various studies, highlighting the complex interplay between gel components and their mechanical properties [40,55,64].

In contrast, HA (*X*_2_) positively influenced the parameter, promoting better shape recovery (e.g., G12). A negative interaction (*X*_1_*X*_2_) between water and HA further underscores the necessity of a balanced formulation. While HA enhances elasticity, excessive water disrupts the gel network, potentially weakening its structural integrity and diminishing the springback potential. This effect is consistent with reports that excessive hydration in biopolymer-based gels can interfere with the crosslinking density, compromising elasticity and mechanical recovery [104,106].

#### 2.3.6. Stringiness (*Y*_10_)

The stringiness (*Y*_10_), a measure of the gel’s stretchability [108], varied from 16.72 ± 0.93 mm (G13) to 26.21 ± 2.42 mm (G12). Water (*X*_1_) negatively affected stringiness, resulting in a less stretchable gel (e.g., G13). This observation aligns with findings in biopolymeric hydrogels, where excessive water weakens polymer entanglements, resulting in diminished mechanical integrity and stretchability [109,110].

Conversely, HA (*X*_2_) positively influenced stringiness, enhancing the stretchability (e.g., G12). This effect underscores HA’s role in forming a cohesive and extensible network, attributed to its high molecular weight and strong water-retaining capacity, which contribute to increased flexibility. Similar trends have been observed in HA-based hydrogels, where higher HA concentrations improve elongation, viscoelasticity, and mechanical adaptability [111,112,113]. These properties make HA-enriched formulations particularly advantageous for mucosal and bioadhesive applications, where an extended retention time and adaptability to dynamic environments are critical for efficacy.

This comprehensive analysis highlights the complex interplay between formulation parameters and textural properties in XTG-HA eutectogels. The water content, while essential for polymer hydration, must be carefully controlled to avoid excessive dilution, which can compromise elasticity and stretchability. Meanwhile, the XTG and HA concentrations should be strategically adjusted to fine-tune the mechanical resilience and extensibility, optimizing the gels for targeted biomedical and pharmaceutical applications. These insights align with recent advances in bio-inspired hydrogels, where tailored polymer interactions enable superior mechanical performance, prolonged retention, and improved bioadhesion [2,6,15,23,36].

### 2.4. In Vitro Release Kinetics Analysis

The rate and extent of drug release from a delivery system are critical factors influencing therapeutic efficacy. This study investigates two key kinetic parameters of drug release from XTG-HA eutectogels: release rate (µg/cm^2^/min^1/2^) (*Y*_11_), representing the steady-state release, and cumulative drug released per surface area at 2 h (µg/cm^2^) (*Y*_12_), reflecting the short-term release. These parameters provide a comprehensive understanding of both the initial burst and sustained release profiles of the formulations.

To assess the drug release kinetics, a flow-through cell apparatus was employed, simulating physiological conditions. Following SUPAC-SS guidelines for semisolid systems, the release rate (*Y*_11_) was determined from the slope of the linear portion of the cumulative release versus square root of time plot, representing steady-state drug diffusion through the membrane, using the Higuchi model [114]. This model quantifies drug diffusion through the gel matrix and across a membrane, mimicking in vivo drug absorption. A sustained, steady-state release rate (*Y*_11_) is essential for maintaining therapeutic drug levels over an extended period, which is particularly crucial for chronic conditions [115]. The cumulative drug released at 2 h (*Y*_12_) provides insight into the initial drug release from the gel, indicating its ability to deliver a therapeutic dose shortly after application [116]. This parameter is particularly relevant when rapid onset of action is desired.

**Figure 5:**
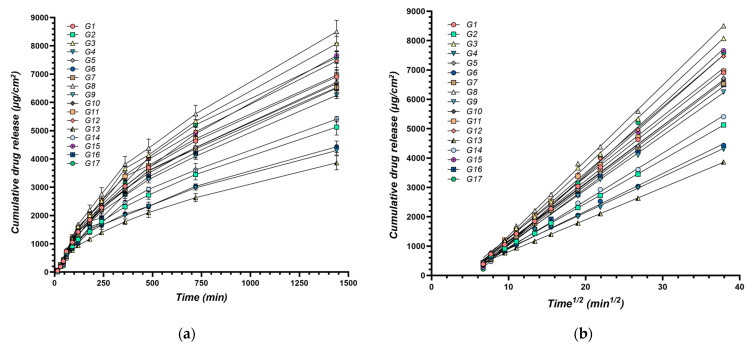
Cumulative amount of IBU diffused through membranes over time: (**a**) Cumulative drug release per surface area (μg/cm^2^) over time (min), *n* = 3; (**b**) Cumulative drug release (μg/cm^2^) plotted against the square root of time (min^1/2^); the continuous line represents the linear fitting of the steady state drug diffusion through the membrane used for calculation of the release rate.

By analyzing these kinetic parameters, this study aims to elucidate the impact of formulation variables on drug release profiles. Understanding these influences can guide the development of XTG-HA eutectogels with tailored drug delivery characteristics, optimizing both the initial burst and sustained release of the therapeutic agent to meet the specific needs of various clinical scenarios. The cumulative amount of IBU diffused through membranes over time for all experimental formulations is presented in Figure 5.

The release rate (*Y*_11_) ranged from 109.4 ± 6.1 µg/cm^2^/min^1/2^ (G13) to 256.6 ± 23.7 µg/cm^2^/min^1/2^ (G8) (Table 1). This range demonstrates the significant impact of formulation variables on the rate at which the active ingredient is released from the gel matrix.

**Figure 6:**
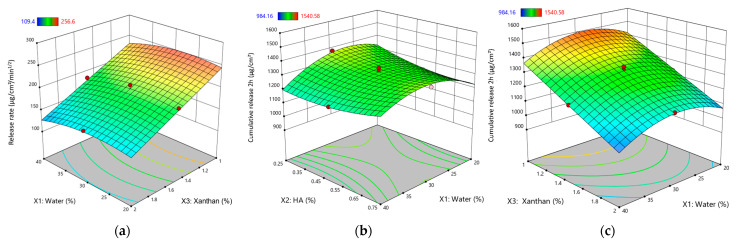
Response surface plots for kinetic parameters of XTG-HA eutectogels: (**a**) Release rate (μg/cm^2^/min^1/2^) as a function of water (%) and XTG (%); (**b**) Cumulative drug release at 2 h (μg/cm^2^) as a function of HA (%) and water (%); (**c**) Cumulative drug release at 2 h (μg/cm^2^) as a function of XTG (%) and water (%). Only graphs including very significant influences (*p* < 0.0001) are depicted.

Increasing the water content had a negative effect on *Y*_11_, decreasing the release rate (Figure 6a). This is attributed to the dilution effect of water, reducing the concentration gradient of the active ingredient and slowing its diffusion through the gel matrix.

XTG exhibited a strong negative influence on *Y*_11_, significantly decreasing the release rate. This aligns with its thickening properties, leading to a denser gel network that hinders the diffusion of the active ingredient.

HA did not have a statistically significant effect on *Y*_11_ (Table 2). While it may contribute to the gel’s structural integrity, its impact on the release rate was not pronounced within the studied formulations.

The positive interaction between water (*X*_1_) and XTG (*X*_3_) suggests that water can mitigate the rate-reducing effects of XTG. This implies that optimizing the balance between these two components is crucial for achieving a desired release rate.

The negative quadratic effect of XTG ($X_{3}^{2}$) indicates that at higher concentrations, its rate-reducing effect becomes even more pronounced. This highlights the importance of carefully controlling the XTG concentration to achieve the desired release profile.

The cumulative release at 2 h (*Y*_12_) varied from 984 ± 81 µg/cm^2^ (G13) to 1541 ± 100 µg/cm^2^ (G8). Similar to its effect on *Y*_11_, water exhibited a negative effect on *Y*_12_, significantly decreasing the cumulative release (Figure 6b,c). This reinforces the understanding that a higher water content leads to a more diluted gel matrix, slowing down the diffusion process and reducing the overall amount of drug released within the given timeframe.

Consistent with its impact on *Y*_11_, XTG significantly decreased *Y*_12_. This further supports its role in forming a dense gel network that hinders the diffusion of the active ingredient, ultimately reducing the cumulative release. While not statistically significant, HA tended to reduce *Y*_12_, suggesting a potential role in controlling the initial burst release. This effect could be attributed to its influence on the gel’s structural integrity and its interaction with the other components.

The positive interaction between water (*X*_1_) and XTG (*X*_3_) indicates that water can offset the cumulative release-reducing effects of XTG, highlighting the importance of their interplay in achieving the desired release profiles.

Interestingly, both HA ($X_{2}^{2}$) and XTG ($X_{3}^{2}$) exhibited positive quadratic effects on *Y*_12_. This suggests that at higher concentrations, their combined influence on the gel’s structure and porosity might lead to an increase in the cumulative release, possibly due to changes in the gel’s network structure or swelling behavior.

To gain deeper insight into the underlying release mechanisms, the data were further analyzed using multiple mathematical models, each reflecting distinct kinetic processes. These included the following:

- Zero-order model: characterizes a constant drug release over time, independent of concentration, typically associated with systems designed for prolonged and uniform drug delivery [117].
- First-order model: assumes a concentration-dependent release, where the rate decreases as the drug is depleted from the formulation, often observed in matrix-based or dissolution-controlled systems [118].
- Higuchi model: describes drug release governed by Fickian diffusion from a homogeneous matrix, applicable to systems where the release is driven by a concentration gradient within the polymer network [119].
- Korsmeyer–Peppas model: a semi-empirical equation that provides insights into the release mechanism by incorporating both diffusion and polymer relaxation effects. The release exponent (*n*) derived from this model helps determine whether the release follows Fickian diffusion, anomalous transport, or erosion-based mechanisms [116].
- Weibull model: a flexible empirical equation that can describe a wide range of release profiles, allowing for differentiation between purely diffusive transport, anomalous release behaviors, and erosion-controlled kinetics [120].

To determine the most appropriate kinetic model for each formulation, the goodness of fit was assessed using statistical parameters such as correlation coefficients (*R*^2^) and the Akaike information criterion (AIC) [121].

The kinetic parameters for drug release from the XTG-HA eutectogels, including correlation coefficients (*R*^2^) and rate constants for each mathematical model, are summarized in Table 3.

The release kinetics of the XTG-HA eutectogels were primarily characterized using the Korsmeyer–Peppas model, with release exponent (*n*) values ranging from 0.507 to 0.638, indicating anomalous (non-Fickian) transport where diffusion is the dominant mechanism, with a minor contribution from polymer relaxation and swelling. This behavior is expected for hydrophilic gel-based matrices, where swelling and gradual polymer erosion contribute to the release process [122]. Additionally, the Higuchi model exhibited consistently high correlation coefficients (*R*^2^ > 0.99) across all formulations, confirming that Fickian diffusion is the predominant release mechanism [116]. Further statistical analysis, including the Akaike information criterion (AIC) and nested F-test comparisons, revealed no significant differences between the two models in terms of overall fit. Consistent with the USP-recommended approach for semisolid formulations, the Higuchi model is preferred as the simplest and most mechanistically relevant method for describing drug release from these eutectogel formulations [118].

These findings highlight the key advantages of incorporating IBU into the XTG-HA eutectogel, particularly in enhancing buccal drug delivery. Beyond enabling controlled and sustained release, the formulation employs multiple mechanisms to improve drug absorption and therapeutic efficacy. First, the inclusion of NADES significantly enhances IBU solubility by disrupting its crystalline structure, thereby increasing the amount of drug available at the absorption site, as already presented elsewhere [7]. Second, the strong mucoadhesive properties of the eutectogel prolong its retention on the mucosal surface, reducing drug loss due to saliva clearance and enabling extended local absorption, as demonstrated by the mucosal retention experiments. Additionally, the controlled release profile, primarily governed by diffusion-based Higuchi kinetics, ensures a steady and sustained drug release, minimizing fluctuations in drug concentration. Finally, the presence of hydrating and permeation-enhancing excipients, such as ChCl and HA, may further facilitate drug permeation across the mucosal epithelium, maintaining a hydrated interface that enhances absorption and bioavailability.

### 2.5. Correlation Analysis of Rheological, Textural, and Kinetic Properties of the Experimental Eutectogel Formulations

In the context of developing eutectic gel systems, it is crucial to understand the interrelationships between the rheological, textural, and kinetic parameters to optimize the formulation for specific biomedical applications. This analysis examines the correlations between the 12 response variables (*Y*_1_ − *Y*_12_) grouped into rheological (*Y*_1_ – *Y*_4_), textural (*Y*_5_ − *Y*_10_), and kinetic (*Y*_11_ − *Y*_12_) categories.

**Figure 7:**
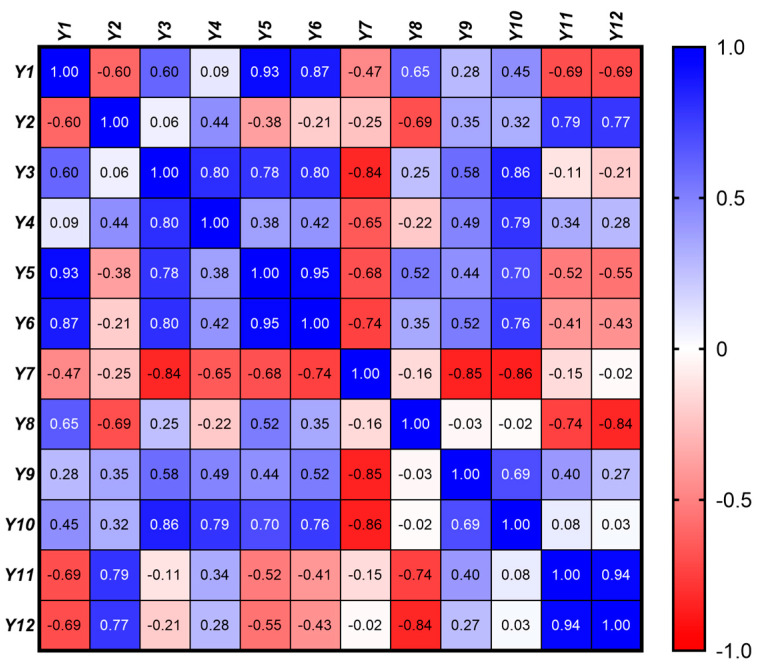
Pearson correlation matrix illustrating the linear relationships among the rheological (*Y*_1_ – *Y*_4_), textural (*Y*_5_ − *Y*_10_), and kinetic (*Y*_11_ − *Y*_12_) properties of the eutectic gel formulations. The color gradient represents the strength and direction of the correlations, with values ranging from −1 (strong negative correlation, red) to +1 (strong positive correlation, blue).

In order to assess the linear relationships between the 12 response variables, a Pearson correlation matrix was generated. The Pearson correlation coefficient, *r*, was calculated for each pair of variables, providing a measure of the strength and direction of their linear relationship (Figure 7). The correlation coefficients range from −1 to 1, where values closer to 1 or −1 indicate stronger positive or negative correlations, respectively, while values near 0 indicate weak or no linear relationship. *p*-values associated with each correlation coefficient were calculated to test the significance of the correlations. A significance level (α) of 0.05 was used, meaning that *p*-values less than 0.05 were considered statistically significant, indicating strong evidence against the null hypothesis (i.e., no correlation). These tests help determine which correlations are likely to be real versus those that could be due to random chance.

A detailed analysis of both intra-group (within rheological, textural, and kinetic properties) and inter-group (between different property groups) correlations was performed, offering insights into the underlying mechanisms that influence gel performance.

#### 2.5.1. Rheological Properties (*Y*_1_ − *Y*_4_)

A significant negative correlation (r = −0.60, *p* = 0.011) between the consistency index (*Y*_1_) and the flow behavior index (*Y*_2_) revealed that gels with a higher viscosity exhibit more pronounced shear-thinning behavior. This suggests that as the consistency of the gel increases, it tends to shear-thin more rapidly, which is characteristic of a more structured gel matrix that readily flows under shear but retains its structure when at rest.

Positive correlations were also found between the consistency index and both *S_thix_* (*Y*_1_ vs. *Y*_3_, *r* = 0.60, *p* = 0.012) and TI (*Y*_1_ vs. *Y*_4_, *r* = 0.60, *p* = 0.0001), implying that more viscous gels exhibit greater thixotropy, as evidenced by their larger hysteresis loop area and higher thixotropy index.

A strong positive correlation was identified between *S_thix_* and *TI* (*Y*_3_ vs. *Y*_4_, *r* = 0.80, *p* = 0.0009). This indicates that gels experiencing more significant structural breakdown during shear (as indicated by a larger hysteresis loop area) also tend to recover their structure more effectively post-shear.

#### 2.5.2. Textural Properties (*Y*_5_ − *Y*_10_)

A very strong positive correlation between hardness and adhesiveness was observed (*Y*_5_ vs. *Y*_6_, *r* = 0.95, *p* < 0.0001), indicating that harder gels tend to be more adhesive. This relationship is likely due to the increased network density, which enhances both their mechanical strength and their ability to adhere to surfaces.

Similarly, a strong positive correlation with cohesiveness (*Y*_5_ vs. *Y*_7_, *r* = 0.80, *p* < 0.0001) suggests that harder gels are also more cohesive. This finding aligns with the expectation that increased gel firmness enhances both its internal bonding strength and overall structural integrity.

The strong correlation between adhesiveness and cohesiveness (*Y*_6_ vs. *Y*_7_, *r* = 0.95, *p* < 0.0001) further highlights the interdependence of these textural properties. Adhesive gels, which stick well to surfaces, are likely to maintain internal cohesion, ensuring that the gel remains intact during application and use.

A positive correlation between cohesiveness and resilience (*Y*_7_ vs. *Y*_8_, *r* = 0.74, *p* < 0.0001) suggests that more cohesive gels also recover better after deformation. This finding is significant as it indicates that cohesive gels, which maintain their internal structure, are also more likely to exhibit good resilience, bouncing back to their original form after being disturbed. The positive correlation between resilience and springiness (*Y*_8_ vs. *Y*_9_, *r* = 0.52, *p* = 0.034) suggests that resilient gels, which recover their shape after deformation, also exhibit good elasticity. This property is particularly important for gels that need to maintain their form and function under dynamic conditions. The correlation between springiness and stringiness (*Y*_9_ vs. *Y*_10_, *r* = 0.69, *p* = 0.002)) indicates that gels with greater springiness are also stringier, likely due to a more elastic and cohesive network that allows the gel to stretch under force. This suggests that gels with higher elasticity are also more likely to exhibit extended behavior under mechanical stress.

#### 2.5.3. Kinetic Properties (*Y*_11_ − *Y*_12_)

A very strong positive correlation between the release rate and cumulative release was observed (*Y*_11_ vs. *Y*_12_, r = 0.94, *p* < 0.0001), indicating that formulations with a higher initial drug release rate also tend to release a larger total amount of drug within the first two hours. This relationship, reflecting the fundamental connection between the speed and extent of drug release, is critical for optimizing drug delivery profiles to achieve the desired therapeutic effect.

#### 2.5.4. Inter-Group Correlations

The strong positive correlations between the consistency index (*Y*_1_) and both hardness (*Y*_5_, *r* = 0.93, *p* < 0.0001) and adhesiveness (*Y*_6_, *r* = 0.87, *p* < 0.0001) reveal that increased viscosity leads to harder and more adhesive gels. This is advantageous for applications requiring shape retention and strong surface adhesion. However, this enhanced mechanical strength comes at the cost of reduced cohesiveness (*Y*_7_, *r* = −0.47, *p* = 0.05), implying a potential trade-off between these properties. Achieving a balance to ensure sufficient cohesiveness for effective application while maintaining adequate hardness and adhesion is crucial.

Furthermore, moderate negative correlations between the consistency index (*Y*_1_) and both the drug release rate (*Y*_11_, *r* = −0.69, *p* = 0.02) and cumulative drug release at 2 h (*Y*_12_, *r* = −0.69, *p* = 0.02) indicate that higher viscosity gels impede drug release. This is attributed to the denser gel matrix hindering drug diffusion. Therefore, balancing viscosity to achieve both adequate mechanical strength and efficient drug release, particularly for applications requiring rapid or sustained release, is critical.

Interestingly, the positive correlations between the flow behavior index and kinetic parameters (*Y*_2_ vs. *Y*_11_, *r* = 0.79, *p* = 0.002 and *Y*_2_ vs. *Y*_12_, *r* = 0.77, *p* = 0.002) suggest that gels exhibiting less pronounced shear-thinning allow for more efficient drug release. This may be due to a less compact network structure facilitating drug diffusion.

While not statistically significant, the negative correlation between the flow behavior index and cohesiveness (*Y*_2_ vs. *Y*_7_, *r* = −0.25, *p* = 0.335) suggests that gels with more pronounced shear-thinning may be less cohesive. This could be due to the weaker internal network structure in shear-thinning gels, compromising their ability to maintain internal cohesion.

On the other hand, the strong negative correlation between *S_thix_* (*Y*_3_) and cohesiveness (*Y*_7_, *r* = −0.84, *p* < 0.0001) suggests that gels experiencing greater energy loss during shear (larger hysteresis loop) are less likely to maintain their internal structure, thus becoming less cohesive. This finding is significant because it highlights the importance of minimizing energy loss during shear to preserve the gel’s cohesiveness, which is crucial for maintaining the gel’s structural integrity during application. The positive correlation between *S_thix_* and hardness (*Y*_3_ vs. *Y*_5_, *r* = 0.78, *p* = 0.0002) suggests that gels with greater thixotropic recovery also tend to be harder. This may be attributed to the structural robustness required to recover from deformation, which contributes to increased gel hardness.

The moderate positive correlation between *TI* (*Y*_4_) and adhesiveness (*Y*_6_, *r* = 0.42, *p* = 0.093), coupled with a moderate correlation with hardness (*Y*_5_, *r* = 0.38, *p* = 0.136), indicates that gels capable of recovering their viscosity after shear are also more likely to maintain their adhesive and structural properties. These findings suggest that enhancing the thixotropy could help improve both the application properties and mechanical stability of the gel. However, the relatively weaker correlations with these textural properties compared to viscosity suggest that while thixotropy contributes to these characteristics, viscosity is a more dominant factor.

Finally, the negative correlations between hardness and the kinetic parameters (*Y*_5_ vs. *Y*_11_, *r* = −0.52, *p* = 0.032 and *Y*_5_ vs. *Y*_12_, *r* = −0.55, *p* = 0.021) indicate that harder gels tend to release drugs more slowly and less efficiently. This is logical, as a firmer gel matrix would hinder drug diffusion. Although not statistically significant, the negative correlations between adhesiveness and drug release (*Y*_6_ vs. *Y*_11_, *r* = −0.41, *p* = 0.099 and *Y*_6_ vs. *Y*_12_, *r* = −0.43, *p* = 0.083) suggest that more adhesive gels might also slow down drug release. This could be due to stronger interactions between the drug and the gel matrix, which hinder the drug’s diffusion out of the gel.

### 2.6. Optimization of XTG-HA-NADES Eutectogels

Analyzing the influence of the formulation factors and of some responses on the characteristics of the studied formulation, a desirability function approach was employed, enabling the simultaneous optimization of these properties by converting each response into a dimensionless desirability score, ranging from 0 (undesirable) to 1 (highly desirable). The overall desirability was then calculated as the geometric mean of the individual desirability scores, providing a comprehensive measure of the formulation’s performance.

The gel should maintain sufficient viscosity to be stable and provide adequate structural support, but it should not be so high that it hinders application or drug release. To achieve this, we targeted a consistency index (*K*) within the range of 50–100 Pa·s^n^.

A flow behavior index (*n*) exceeding 0.25 was deemed optimal to ensure pronounced shear-thinning without compromising gel cohesiveness.

Furthermore, the gel’s ability to recover its structure post-application, crucial for sustained drug release, was addressed by targeting a hysteresis loop area between 2000 and 4000 Pa·s^−1^. This moderate range reflects a balance between desirable shear-thinning and adequate structural recovery. The optimization also targeted maximizing the thixotropy index (*Y*_4_), calculated as the hysteresis loop area normalized to the forward curve area. This ensured robust structural regeneration following shear, contributing to the gel’s overall stability and effectiveness.

Moderate hardness (>0.8 N) was targeted; the gel should have enough firmness to provide a protective barrier but still be soft enough for comfortable application and adhesion to the mucosal surface.

Maximizing both adhesiveness (*Y*_6_) and cohesiveness (*Y*_7_) was paramount to ensure prolonged residence time at the mucosal surface and prevent premature gel breakdown. High adhesiveness ensures the gel firmly attaches to the mucosal surface, maximizing the duration of drug contact and, consequently, absorption. Complementing this, high cohesiveness guarantees the gel maintains its structural integrity throughout the application process and while residing on the mucosa. This prevents premature fragmentation or breakdown, which could compromise the gel’s efficacy. Maximum values for both were included in the optimization algorithm.

Moderate resilience was targeted, allowing the gel to recover its shape after the deformation that inevitably occurs during application. This ensures the gel can adapt to the contours of the mucosal surface while maintaining its structural integrity. However, excessive springiness is undesirable because if the gel is too elastic, it could detach from the mucosa, undermining its effectiveness. However, for both these parameters, the obtained range in the experimental design was deemed appropriate, and no further restrictions were applied.

Minimized stringiness was targeted, in order to prevent the gel from being too sticky or uncomfortable to apply, which could cause issues during administration and reduce patient compliance.

Finally, the kinetics of drug release from the gel matrix are paramount. A controlled release profile, characterized by a high release rate, is essential for sustained and effective therapeutic action. Additionally, a maximized cumulative release within the initial hours is advantageous for achieving a rapid onset of action, which is often critical in mucosal drug delivery, especially for acute conditions.

**Figure 8:**
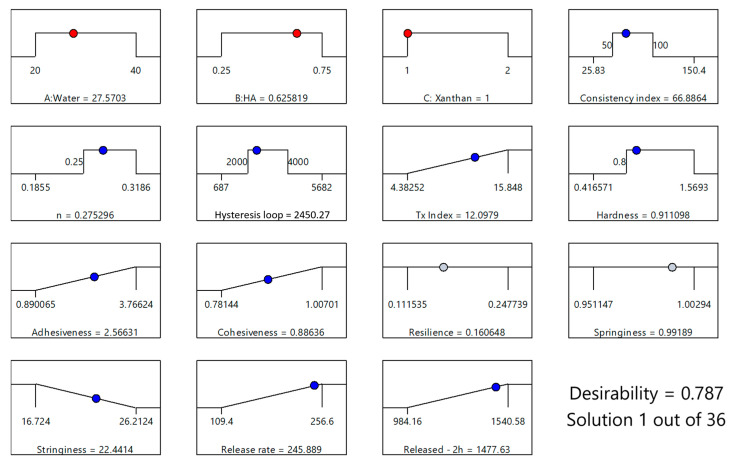
Optimization plot for the XTG-HA-NADES-IBU formulation, based on the maximum value for the desirability function, showing the selected levels of independent variables (X1: Water, X2: HA, X3: XTG) in red and the predicted responses (consistency index, *n*, hysteresis loop, thixotropy index, hardness, adhesiveness, cohesiveness, resilience, springiness, stringiness, release rate, and cumulative release at 2 h). The blue circles indicate responses used in the optimization, while the gray circles indicate that no restrictions were imposed on the respective responses.

The optimized formulation, with a desirability score of 0.787, consisted of 27.57% water, 0.63% HA, and 1% XTG and contained 2.5% IBU (Figure 8). This specific composition was identified as the most favorable balance between the various critical parameters, ensuring both optimal physical properties and effective drug delivery performance. This formulation (coded XTG-HA-NADES-IBU) was prepared in triplicate and studied for its rheological, textural, and kinetic properties. Each experimental evaluation was performed in duplicate, resulting in a total of six determinations for each response.

The observed experimental responses for XTG-HA-NADES-IBU were close to the theoretically estimated ones (Table 4).

### 2.7. Impact of the NADES Presence on the Rheological, Textural, and Microstructural Properties of the Optimized Eutectogel Formulation

**Figure 9:**
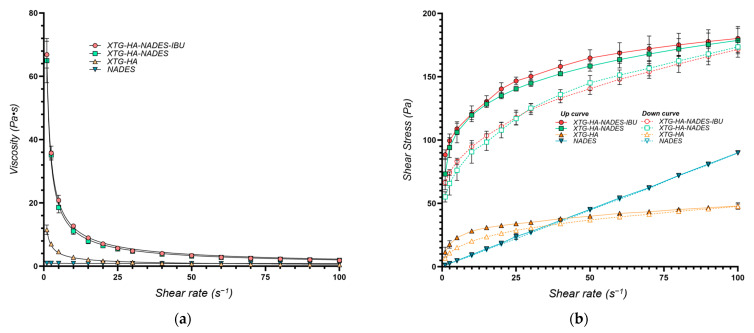
Rheological properties of the optimum eutectogel formulation (XTG-HA-NADES-IBU) and its components at 37 °C represented as (**a**) viscosity (Pa·s) vs. shear rate (s^−1^) and (**b**) shear stress (Pa) vs. shear rate (s^−1^).

The viscosity and shear stress profiles (Figure 9) illustrate that the presence of NADES substantially enhances the consistency index of the gels, with both the gel composed of HTG, HA, and NADES (coded XTG-HA-NADES) and the one containing 2.5% IBU (XTG-HA-NADES-IBU) exhibiting similar and significantly higher viscosity compared to the XTG-HA gel base. The presence of NADES facilitates the formation of a more structured network within the gel matrix, superior to the polymeric gel base alone.

The *K* values are similar for the two eutectogel formulations (XTG-HA-NADES and XTG-HA-NADES-IBU), whereas the gel base and the NADES system are significantly less viscous (Table 5). Interestingly, while the flow behavior index for the NADES is very close to 1, indicating a near-Newtonian flow, its combination with the XTG-HA base leads to a significant increase in the shear-thinning behavior of the gel base, with *n* decreasing from 0.3604 to 0.2407.

The findings further confirms that NADES alone lacks the necessary viscosity and shear-thinning properties for effective gel formulation, emphasizing the importance of combining it with XTG-HA to achieve desirable rheological characteristics.

In terms of texture properties, the XTG-HA formulation exhibits significantly lower hardness compared to both the XTG-HA-NADES and XTG-HA-NADES-IBU formulations. The addition of NADES and IBU substantially increases the hardness of the gel, with no significant difference between the XTG-HA-NADES and XTG-HA-NADES-IBU formulations.

**Figure 10:**
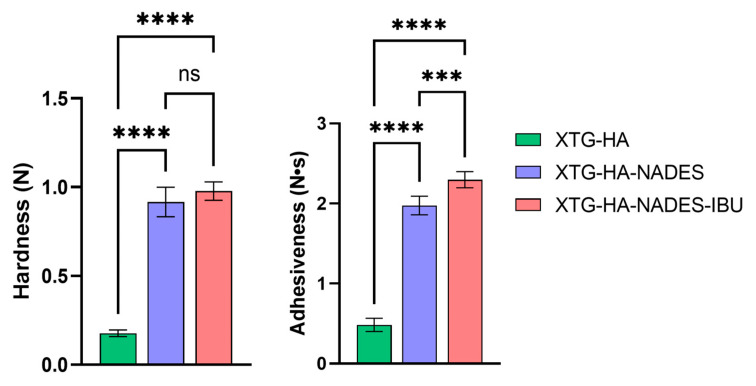
Comparison of textural properties—hardness (N) and adhesiveness (N·s)—among the three formulations: XTG-HA, XTG-HA-NADES, and XTG-HA-NADES-IBU (*** *p* < 0.001, **** *p* < 0.0001, ns: not significant).

Similar to hardness, the XTG-HA formulation shows much lower adhesiveness. Both the XTG-HA-NADES and XTG-HA-NADES-IBU formulations exhibit significantly higher adhesiveness, with XTG-HA-NADES-IBU showing the highest value (Figure 10). This suggests that the presence of NADES and IBU enhances the adhesive properties of the gel, which is critical for its performance on mucosal surfaces.

All other texture property differences between XTG-HA, XTG-HA-NADES, and XTG-HA-NADES-IBU are not statistically significant and are not depicted in the figure.

**Figure 11:**
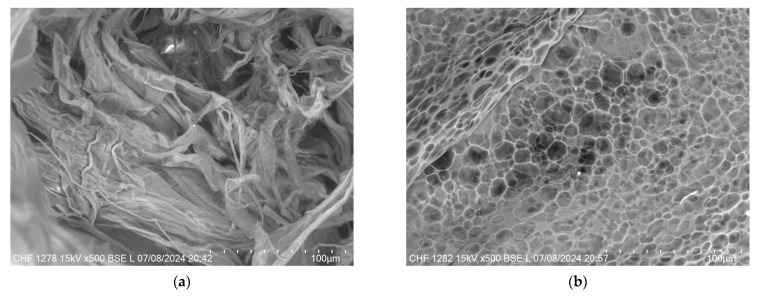
SEM micrographs of (**a**) XTG-HA and (**b**) XTG-HA-NADES.

Figure 11 provides a comparative view of the microstructural differences between the XTG-HA and XTG-HA-NADES formulations observed through scanning electron microscopy (SEM). In Figure 11a, the micrograph of XTG-HA reveals a fibrous network, where the XTG and HA form closely packed layers, contributing to its mechanical strength and gel-like consistency. In contrast, Figure 11b displays the microstructure of XTG-HA-NADES, which shows a more porous, honeycomb-like structure. This porous architecture is indicative of the influence of the NADES component, which disrupt the tightly packed fibers, leading to a more open structure.

The resulting eutectogels were developed through physical interactions, including hydrogen bonding, polymer entanglement, and electrostatic interactions, without covalent modifications. XTG affects the hydrogen bonding network of the NADES system, which is crucial for eutectogel formation [23]. This interaction is similar to the formation of xanthan gum-based hydrogels, where water addition and annealing are necessary [23,36,59]. Rheological analysis confirmed a shear-thinning and thixotropic profile, characteristic of physically crosslinked hydrogels [123]. The high bioadhesion and cohesiveness suggest strong non-covalent interactions with mucosal surfaces [124].

### 2.8. DSC Measurements

**Figure 12:**
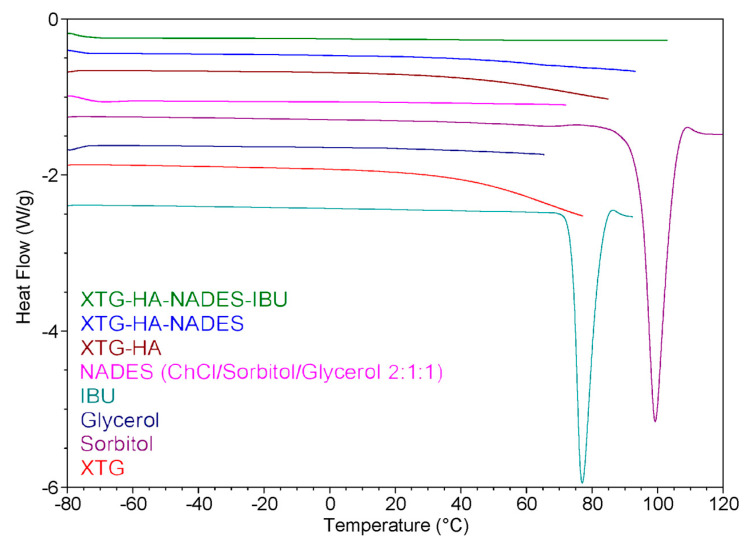
Differential scanning calorimetry (DSC) heating curves of various components and eutectogel formulations.

The DSC heating curves for the prepared eutectogels, NADES, and the other individual components of the gel systems are presented in Figure 12. The DSC thermogram suggests that the incorporation of IBU into the eutectogel matrix (XTG-HA-NADES) results in significant interactions between the drug and the gel components. These interactions likely lead to a change in the crystalline state of IBU, as evidenced by the absence of the endothermic peak corresponding to IBU melting from the XTG-HA-NADES-IBU curve. This behavior is consistent with the improved solubility and dispersion of the drug within the gel matrix.

Analysis of the curves corresponding to the NADES-containing formulations provides insights into the role of water in these systems. According to the literature [125], in polysaccharide-based gels, water can exist in different forms: free water, freezing bound water, or non-freezing bound water. Non-freezing bound water, which does not show any phase transition in the temperature range of −73 to 0 °C, is tightly bound to the polysaccharide chains and is crucial for the stability and structure of the gel [126].

Both the XTG-HA-NADES and XTG-HA-NADES-IBU formulations do not show significant endothermic peaks below 0 °C in the DSC curve. This suggests that in these samples, the water is predominantly present as non-freezing bound water. The absence of a distinct melting peak at 0 °C indicates there is little to no free water present, which would typically exhibit a phase transition similar to pure water. It is important to note that water can also act as an integral component of the supramolecular hydrogen-bond network within the NADES structure, further contributing to its bound state [127,128].

Non-freezing bound water ensures that the gel retains its mechanical integrity and adhesive properties even when exposed to the dynamic environment of the buccal cavity [129]. This is particularly important in buccal formulations, where the gel must adhere to the mucosal surface and resist immediate washout by saliva. This finding is consistent with the observations of Xia et al. on polysaccharide-based eutectogels, which indicated that the signals of free water in the thermograms appeared only at water contents greater than 60% [36]. Up to 80% water content, non-freezing water still accounted for the majority of water in the systems.

### 2.9. Swelling Behavior

**Figure 13:**
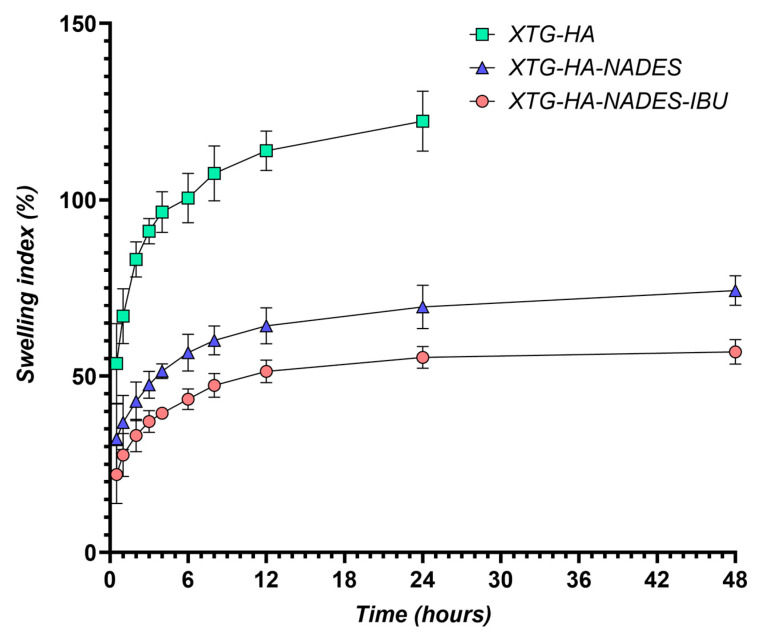
Swelling index (%) of XTG-HA, XTG-HA-NADES, and XTG-HA-NADES-IBU formulations over 48 h in simulated saliva (pH 6.8) at 37 °C.

The swelling behavior of the hybrid eutectogels was monitored over a 48 h interval to evaluate their ability to absorb water while retaining structural integrity (Figure 13). The XTG–HA formulation exhibited the highest swelling index, surpassing 100% within the first few hours and continuing to increase up to nearly 150% at later timepoints, while maintaining the integrity only in the first 24 h of the experiment.

This pronounced swelling can be attributed to the abundance of hydrophilic groups in xanthan gum and hyaluronic acid, which allow for extensive hydrogen bonding with water [130,131,132]. Similar high swelling levels have been widely reported in the literature for polysaccharide-based hydrogels, where hydroxyl and carboxyl moieties facilitate water uptake and network expansion [133,134].

In contrast, the gels containing the NADES component (XTG–HA–NADES) displayed a notably lower swelling index, stabilizing around 60–70%. Introducing the eutectic mixture of choline chloride and sugar alcohols appears to moderate water penetration by creating additional hydrogen-bonding interactions within the gel matrix [135,136]. Consequently, there is less available free volume for external water to diffuse into, thus limiting excessive swelling [137]. The further incorporation of IBU (XTG–HA–NADES–IBU) led to an even more modest increase in swelling over time, ultimately plateauing around 40–50%. In addition to the NADES-mediated structuring, the partially hydrophobic character of IBU likely impedes water diffusion, effectively reducing the gel’s net swelling capacity [138].

These findings align with previous studies on NADES-enriched hydrogels, which commonly show a diminished swelling response compared to their aqueous gel counterparts [138]. The mechanism centers on the high proportion of bound (or “non-freezing”) water within NADES-rich networks [139]. Because the hydroxyl and quaternary ammonium groups in the choline–sugar alcohol mixture can strongly interact with both the polymer chains and water molecules, less water remains unbound and free to migrate in or out of the network [140]. The literature corroborates that a higher fraction of non-freezing bound water generally translates into lower swelling yet enhanced mechanical stability, as the gel matrix does not drastically expand or collapse upon hydration [82]. This non-freezing water-dominated regime ultimately underlies both the improved dimensional stability and the controlled swelling profiles seen in the hybrid NADES–polysaccharide gels [129].

Moreover, the presence of a robust NADES–polysaccharide network can shift or broaden the glass-transition region of the gels. This structural feature, in turn, helps maintain a cohesive matrix even under conditions that would typically promote extensive swelling [141]. Taken together, the reduced swelling observed in XTG–HA–NADES and XTG–HA–NADES–IBU points to an improved hydrogel integrity and more controlled water uptake, with most of the absorbed water being in the non-freezing state. Such moderate and stable swelling profiles are particularly beneficial for formulations designed for mucosal application, where excessive swelling could compromise adhesion or drug release consistency [142].

Overall, these results demonstrate that introducing a ChCl–sugar alcohol eutectic mixture into polysaccharide-based hydrogels effectively curbs over-swelling, yielding a more dimensionally stable and robust delivery platform. The strong interactions among the NADES species, polymer functional groups, and water molecules underpin the enhanced stability of the eutectogels upon dilution and hydration, paving the way for improved performance in buccal or similarly moist environments.

### 2.10. Mucosal Ex Vivo Residence Time

The ex vivo mucosal residence time of the tested formulations demonstrated significant differences, highlighting the impact of NADES incorporation and IBU loading on bioadhesion. The XTG-HA formulation exhibited a residence time of 72.3 ± 4.2 min, suggesting a moderate mucoadhesive potential, likely driven by the hydrogen bonding and viscosity of XTG and HA. However, the addition of a NADES in XTG-HA-NADES markedly enhanced mucosal retention, extending the residence time to 182.5 ± 17.8 min. This significant increase suggests that NADES strengthens the polymer–mucus interactions, potentially by improving hydration, enhancing polymer entanglement, and increasing adhesive forces with mucosal glycoproteins.

Interestingly, the incorporation of IBU into the NADES-based formulation (XTG-HA-NADES-IBU) resulted in a slight decrease in the mucosal residence time (176.7 ± 23.1 min), though this difference was not statistically significant. This suggests that while IBU does not drastically alter the bioadhesive properties of the formulation, it may induce minor structural changes that slightly reduce polymer–mucus interactions [143]. The observed decrease might be attributed to competitive interactions between the IBU and mucosal components, which could marginally affect the gel’s adhesion and swelling behavior [144].

Overall, these findings confirm that NADES plays a crucial role in prolonging mucosal retention, making these formulations promising candidates for sustained drug delivery applications. The significantly extended residence time of NADES-containing eutectogels suggests their potential for enhancing drug absorption and therapeutic efficacy in mucosal drug delivery systems. Further in vivo investigations will be necessary to validate these findings and assess their impact on drug release kinetics and bioavailability.

### 2.11. Antimicrobial Activity

While the formulation does not contain known antimicrobial agents, testing for antimicrobial activity is a crucial step in understanding its full range of properties, ensuring safety, and potentially uncovering new avenues for therapeutic applications. NADESs, in particular, are known for their solubilizing capabilities and potential to disrupt microbial cell membranes, which could impart antimicrobial properties to the formulation even in the absence of conventional antimicrobials [145], with more and more recent studies suggesting their intrinsic antimicrobial potential [146,147,148].

The oral cavity serves as a reservoir for the colonization and infection of systemic organs by harmful microorganisms. It is known that bacterial accumulation and biofilm development in the oral cavity are influenced by a number of variables, including age, tooth eruption, hormone fluctuations, active illness, and dental hygiene [149]. *Bacillus cereus* is a Gram-positive strain that is commonly found in the upper respiratory tract. It appears that immunocompromised patients have a very widespread oral cavity invasion of *B. cereus* because the bacteria can enter the oral cavity through contaminated food or the inhalation of spores [150]. Considered as transitory constituents of the oral microbiome, enterococci can induce a range of systemic and oral infections. In a healthy oral cohort, Komiyama et al. [151] assessed *Enterococcus* spp. and their virulence features, including antibiotic resistance, due to a lack of data on oral enterococcal prevalence. Enterococci—such as *E. faecium*—have become significant pathogens, especially in nosocomial environments [152]. Enterobacteriaceae was the family of Gram-negative bacteria that predominated in oral cavity infections overall. The most common species was *E. coli*, accounting for 53.06% of the infections, followed by *K. pneumoniae*, accounting for 28.57%, according to Saad Alghamdi [153]. Saliva from vulnerable individuals and the environment can transmit *Enterobacteriaceae*, which may find a good environment in the oral cavity. Dietary practices and food habits have a significant impact on the complexity of the oral cavity population.

The eutectogel’s components had no effect on the *B. cereus* and *K. pneumoniae* strains, although the chosen strains were sensitive to a concentration of 25 mg/mL XTG-HA-NADES-IBU (Table 6). The NADES, XTG-HA, and XTG-HA-NADES were found to have MIC values greater than XTG-HA-NADES-IBU for *E. faecium*. Our results correlate with those obtained by Chan et al. [154], noting that the *B. cereus* strain was sensitive to IBU, while *K. pneumoniae* was resistant (Table 5). The antimicrobial activity of IBU against the *E. faecium* strain has not been reported until now.

For any of the chosen strains, no variation showed bactericidal effects.

**Figure 14:**
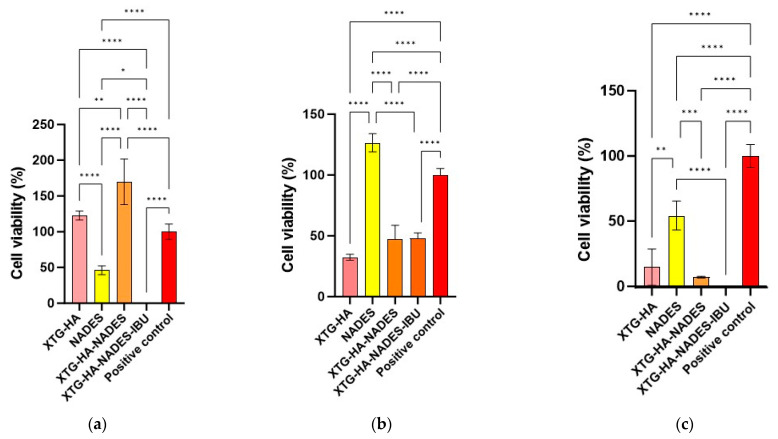
The viability of bacterial cells in the presence of 50 mg/mL formulations diluted in culture medium (TSB) by the spectrophotometric method: (**a**) *B. cereus;* (**b**) *E. faecium;* (**c**) *K. pneumoniae* (* *p* < 0.05, ** *p* < 0.01, *** *p* < 0.001, **** *p* < 0.0001).

To evaluate the degree of microbial growth reduction, microbial viability was evaluated in the presence of 50 mg/mL of the samples. Thus, from Figure 14a, it can be seen that the microbial reduction was significantly lower for the eutectic mixture (NADES) compared to the XTG-HA blank gel base (*p* < 0.0001) and compared to the positive control (*p* < 0.0001), while for XTG-HA-NADES-IBU, the microbial viability decreased significantly compared to XTG-HA base (*p* < 0.0001), the positive control (*p* < 0.0001), and the NADES mixture (*p* < 0.05).

In the case of the *E. faecium* strain, it was observed that the NADES is less active than the gel base (*p* < 0.01). It can be highlighted that the IBU-containing gel (XTG-HA-NADES-IBU) is significantly more active than the NADES (*p* < 0.0001), which may suggest that IBU and the gel components act synergistically (Figure 14b). The NADES proved to not be effective against the *K. pneumoniae* strain (Figure 14c). It can be observed that the differences between the IBU gel and the blank gel base are not significant (*p* > 0.05), while the IBU gel compared to the positive control significantly reduced the microbial viability (*p* < 0.0001).

The better antimicrobial activity against Gram-positive bacteria (*B. cereus* and *E. faecium*) is primarily due to the structure of the cell wall consisting of peptidoglycans, giving it a porous appearance that allows ibuprofen to penetrate and disrupt cellular processes more easily [155]. Additionally, Gram-negative bacteria (*K. pneumoniae*) have an outer membrane that contains lipopolysaccharides (LPSs), which serves as a robust barrier against the penetration of many substances [156]. Another aspect that contributes to the resistance of Gram-negative bacteria is the presence of porins in the outer membrane, which regulate the entry of molecules [157]. IBU has a hydrophobic character that limits its access through porins, thereby reducing its effectiveness [158]. Additionally, Gram-negative bacteria often possess efflux pumps that actively expel harmful substances, including antimicrobials, from the bacterial cell [159]. This can reduce the intracellular concentration of IBU, making it less effective. This structural complexity is the main reason why the final formulation exhibits better antimicrobial activity against Gram-positive bacteria.

After the gels were in direct contact for 0 (T0) and 24 h (T24), respectively, at 37 °C, the capacity to prevent bacterial growth was assessed using the colony counting method in order to further compare the antibacterial qualities. 

**Figure 15:**
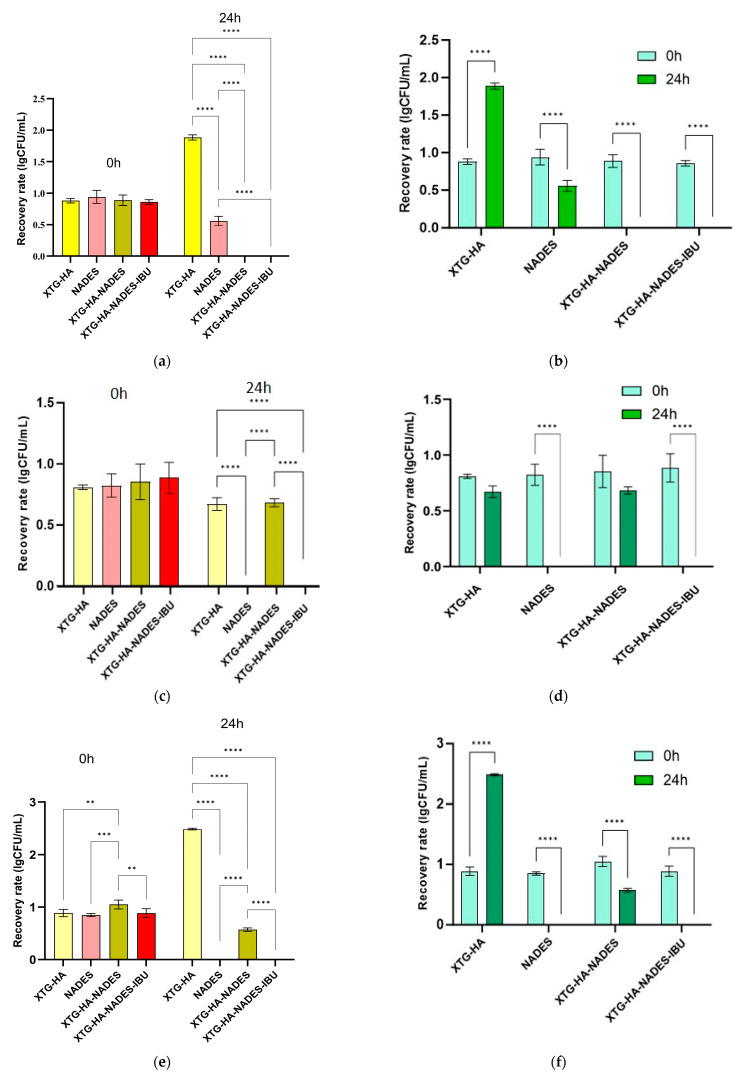
CFU/mL recovery rate at initial contact time (0 h) and after 24 h of incubation at 37 °C for evaluation of XTG-HA, NADES, XTG-HA-NADES, and XTG-HA-NADES-IBU for antimicrobial behavior against (**a**,**b**) *B. cereus*; (**c**,**d**) *E. faecium*; (**e**,**f**) *K. pneumoniae*. The comparative analysis was carried out both for the 4 formulations specific to the time intervals (left) and the differences between the incubation times (right) for each sample (* *p* < 0.05, ** *p* < 0.01, *** *p* < 0.001, **** *p* < 0.0001).

The findings displayed in Figure 15a demonstrate that each combination of eutectogel (either with or without IBU) exhibited a 100% inhibitory effect on *B. cereus* growth, demonstrating a statistically significant difference from the XTG-HA control group, which utilized only the polymer-based hydrogel, without NADES or IBU. Thus, a gel with remarkable antibacterial qualities was produced as a result of the synergistic action of IBU, the basic components, and the eutectic mixture’s intrinsic antimicrobial activity. When comparing T24 for NADES and NADES-containing gels to the initial inoculation (T0), the recovery rate (Figure 15b) was considerably lower (*p* < 0.0001).

It Is evident from Figure 15c that the recovery rate for the XTG-HA-NADES-IBU gel was much lower than that of the XTG-HA-NADES gel; the eutectic combination was mostly responsible for this 100% inhibitory impact. The degree of recovery for the NADES and XTG-HA-NADES-IBU gels was significantly reduced (*p* < 0.0001) when compared to the initial contact time (Figure 15d).

With the exception of the XTG-HA gel base, a considerable drop in the degree of recovery was seen in the case of *K. pneumoniae* even from time T0 (Figure 15e). For all the NADES-containing formulations (NADES, XTG-HA-NADES, and XTG-HA-NADES-IBU), the reduction was significant between T0 and T24 (Figure 15f).

**Figure 16:**
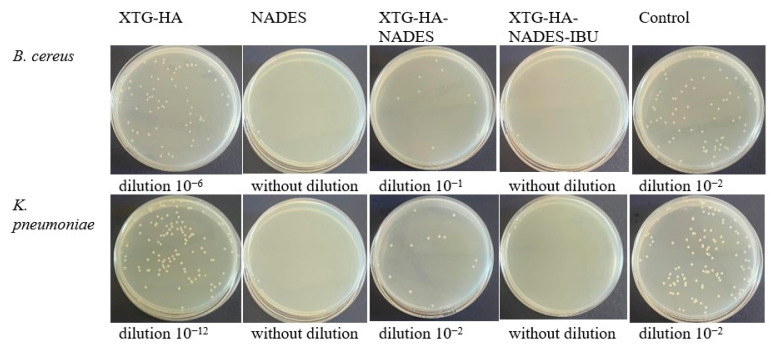
Quantification of CFU/mL after 24 h of eutectogel contact with *B. cereus* and *K. pneumoniae* suspensions (10^5^ CFU/mL).

The results obtained for the *B. cereus* and *K. pneumoniae* strains corresponding to the dilutions made for the CFU/mL evaluation are presented in Figure 16.

Sugar-based NADESs exhibit moderate to weak antimicrobial activity, and the antimicrobial activity of NADESs depends on the number of hydroxyl groups and their acidic character [160]. Jurić et al. [161] found that pure NADESs containing sugar and alcohol as hydrogen donors did not have an antimicrobial effect against *E. coli* and *S. aureus*, a similar effect to that observed in our study against *B. cereus* and *K. pneumoniae* strains. Thus, the selection of HBD species seems to be the key to the antimicrobial effect of pure NADES. Thus, the majority of studies utilizing eutectogels contain compounds for which antimicrobial/microbicidal activity is known (chlorhexidine [162], curcuminoid [163], plant extracts [161,164,165,166]).

On the other hand, IBU, known for its moderate to weak antimicrobial effects, is recommended for use in combination with a well-known antimicrobial agent. The use of IBU in this study led to an enhancement of the antimicrobial activity of the final formulation. According to Obad et al. [167], IBU exhibits antimicrobial activity against both bacterial and fungal strains, but the evaluation was only qualitative. In an in silico study [168], it was found that IBU interacts with proteins associated with both cellular and metabolism processes, turning avirulent. Al-Janabi et al. [169] conducted an in vitro study to evaluate the effectiveness of IBU against Gram-positive and Gram-negative bacteria, namely, *S. aureus*, *B. subtilis*, *E. coli*, *E. aerogenes*, *E. cloacae*, *S. typhi*, and *P. yeei*. IBU showed the best activity against *S. aureus* and *P. yeei*, with an MIC value 1.2 mg/mL, while *Enterobacter* spp. strains proved to be resistant. Thus, we can consider that the antimicrobial effect was generated by a synergistic effect between the sugar bases of NADES and IBU.

These formulations are beneficial in treating localized infections in the oral cavity, such as periodontal pockets or aphthous ulcers, where the dual action of IBU in reducing inflammation and limiting microbial growth helps manage symptoms.

### 2.12. In Vivo Evaluation of Anti-Inflammatory Activity

Two distinct inflammatory stimuli were employed in this study to induce paw edema in the rat model: λ-carrageenan (car) and kaolin (cao). These agents were selected due to their distinct mechanisms of action in triggering inflammatory responses, allowing for a more comprehensive evaluation of the anti-inflammatory properties of the tested formulations.

λ-Carrageenan is a sulfated polysaccharide derived from red seaweed that is widely used to induce acute inflammation in experimental settings. Upon injection, carrageenan triggers a biphasic inflammatory response [170]. The early phase, lasting up to 2 h, is primarily mediated by the release of histamine, serotonin, and bradykinin, leading to vasodilation and increased vascular permeability [171]. The late phase, which peaks between 3 to 5 h post-injection, is characterized by the production of prostaglandins, leukotrienes, and reactive oxygen species, primarily driven by the infiltration of neutrophils and macrophages into the site of inflammation.

Kaolin, on the other hand, is a hydrated aluminum silicate clay mineral that induces a delayed and prolonged inflammatory response primarily mediated by the activation of the complement system and the subsequent release of pro-inflammatory cytokines [172]. Upon injection, kaolin activates the alternative complement pathway, leading to the generation of complement fragments such as C5a, a potent chemoattractant for neutrophils. The recruited neutrophils release a cascade of inflammatory mediators, including cytokines such as IL-1β, IL-6, and TNFα, further amplifying the inflammatory response. This cytokine-driven inflammatory response typically peaks between 6 and 24 h post-injection [171,173].

By employing both carrageenan and kaolin-induced paw edema models, this study aimed to evaluate the efficacy of the IBU formulations against a broader spectrum of inflammatory mediators, encompassing both early-phase mediators such as histamine and prostaglandins, as well as late-phase mediators such as pro-inflammatory cytokines. This approach provides a more comprehensive assessment of the anti-inflammatory potential of the tested formulations.

**Figure 17:**
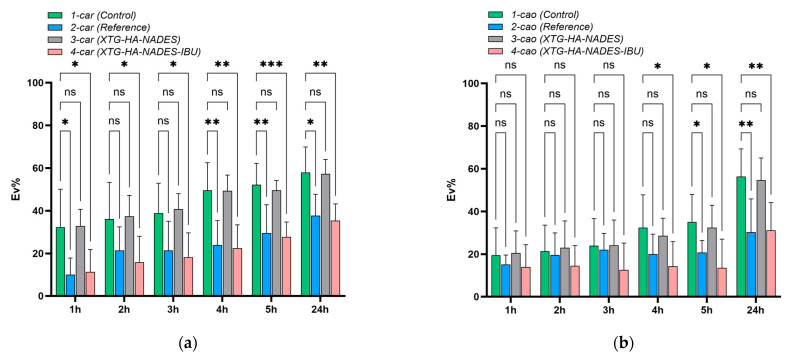
Anti-inflammatory effects of different gel formulations on (**a**) λ-carrageenan and (**b**) kaolin-induced paw edema in rats (* *p* < 0.05, ** *p* < 0.01, *** *p* < 0.001, ns: not significant).

Both the λ-carrageenan- and kaolin-induced inflammation models demonstrate that the XTG-HA-NADES-IBU formulation provides a significant anti-inflammatory effect, particularly in the mid to late phases of the inflammatory response (Figure 17).

In the λ-carrageenan model, the XTG-HA-NADES-IBU formulation demonstrated significant reductions in paw edema across multiple time points, including the early (1–3 h) and mid to late (4–24 h) phases of inflammation (Figure 17a). The significant differences observed between XTG-HA-NADES-IBU and the control group during the early hours, particularly at 1, 2, and 3 h, suggest that the XTG-HA-NADES-IBU gel has a rapid onset of action, effectively mitigating the initial inflammatory response induced by λ-carrageenan. Additionally, the formulation maintained its efficacy throughout the later phases, matching and even outperforming the commercial IBU gel (group 2-car) in reducing inflammation.

In the kaolin-induced model (Figure 17b), there were no significant differences in the Ev% among the treatment groups compared to the control in the early phase of inflammation (1–3 h). This suggests that in the early phase of inflammation, the kaolin-induced response was similar across all groups, and the treatments did not significantly affect the edema formation. However, the XTG-HA-NADES-IBU gel demonstrated a significant anti-inflammatory effect starting from 4 h post-inflammation induction (*p* < 0.05), which was sustained at 24 h (*p* < 0.01). This indicates that the XTG-HA-NADES-IBU gel effectively reduces kaolin-induced inflammation, particularly in the later stages, where cytokine-mediated responses dominate. A similar trend was observed for the commercial IBU gel (group 2-cao), which also significantly reduced edema compared to the control from 5 h onwards, with no significant differences between the XTG-HA-NADES-IBU gel and the commercial IBU gel, indicating comparable efficacy in the later stages of inflammation, despite the XTG-HA-NADES-IBU gel containing only 2.5% IBU, compared to the 5% IBU in the commercial gel. This suggests that the XTG-HA-NADES-IBU formulation may offer a more efficient delivery mechanism for IBU, enhancing its anti-inflammatory effects even at a lower concentration.

## 3. Conclusions

This study successfully developed and characterized a NADES-based eutectogel for buccal drug delivery, demonstrating its multifunctional potential in enhancing drug solubility, prolonging mucosal adhesion, and providing sustained anti-inflammatory and antimicrobial effects. The combination of xanthan gum (XTG), hyaluronic acid (HA), and a choline chloride-based NADES resulted in a formulation with shear-thinning behavior, high mucoadhesion, and extended ex vivo residence time, ensuring prolonged retention at the administration site.

The Higuchi-governed drug release profile confirmed sustained ibuprofen delivery over 24 h, while in vivo anti-inflammatory studies demonstrated a rapid onset of action and prolonged efficacy up to 24 h, comparable to a commercial ibuprofen gel, despite a lower drug concentration (2.5% vs. 5%). The formulation also exhibited potent antimicrobial activity, reducing Gram-positive bacterial viability by 100% upon direct contact, with a minimum inhibitory concentration (MIC) of 25 mg/mL.

These findings position NADES-based eutectogels as a promising platform for buccal drug delivery, particularly for inflammatory conditions with a bacterial component. Future research should focus on in vivo pharmacokinetic studies, long-term stability assessments, and clinical validation to further explore the therapeutic potential and translational feasibility of these formulations.

## 4. Materials and Methods

### 4.1. Materials

Choline chloride ≥ 98% (Sigma-Aldrich, Saint Louis, MO, USA), D-(−)-Sorbitol ≥96%, high purity (VWR Chemicals, Leuven, Belgium), and glycerol ≥ 99% (Sigma-Aldrich, Saint Louis, MO, USA) were used for NADES preparation.

Ibuprofen powder (≥98%) was purchased from Sigma-Aldrich (Saint Louis, MO, USA). Xanthan extra pure (Carl Roth GmbH & Co, Karlsruhe, Germany) and hyaluronic acid sodium salt (Fagron, Rotterdam, The Netherlands) were used as gelling agents.

Ultrapure water (resistivity of 18.2 MΩ·cm at 25 °C and a total organic carbon (TOC) content below 5 ppb) was produced using a Milli-Q EQ 7008 water purification system (Merck Millipore, Burlington, MA, USA).

### 4.2. Preparation of NADES-Based Hybrid XTG-HA Eutectogels

#### 4.2.1. Selection of the NADES System

The selection of the NADES system used for the formulation of eutectogels was driven by an extensive screening study aimed at investigating the solubility of IBU in various eutectic systems, described elsewhere [7]. The evaluation led to the selection of two eutectic systems that exhibited superior solubility characteristics for IBU: a choline chloride-based system, specifically ChCl/sorbitol/glycerol in a 2:1:1 molar ratio, which achieved a solubility of 54 mg IBU/g eutectogel, and a menthol-based system, i.e., menthol/oleic acid, with an impressive solubility of 319 mg/g.

In the present study, which focuses on the development of bioadhesive gels for mucosal administration—particularly for buccal and oral applications—the ChCl/sorbitol/glycerol 2:1:1 NADES was selected as the eutectic system of choice, since it provides a better balance of solubility, biocompatibility, taste masking, and adhesive properties, making it well-suited for effective and patient-friendly mucosal drug delivery.

#### 4.2.2. Preparation of NADES

Ternary NADES was synthesized using choline chloride, D-(-)-sorbitol, and glycerol in a 2:1:1 molar ratio. Choline chloride was dried under vacuum at 50 °C for at least 24 h prior to use and stored in an airtight container. Sorbitol was finely ground using a mortar and pestle to ensure homogeneity.

The components were weighed and combined in a clean, dry, screw-cap glass container within a glovebox to maintain an anhydrous environment. The mixture was then heated to 75 °C and stirred continuously at 1000 rpm for 2 h using a ThermoMixer C system equipped with a suitable thermo-block (Eppendorf, Hamburg, Germany). This process yielded a clear, homogenous liquid, confirming the formation of the DES. The prepared NADESs were stored in airtight vials within a desiccator until further use.

#### 4.2.3. Preparation of Eutectogels

IBU, the model drug, was gradually added to the NADES at 75 °C under continuous stirring (100 rpm) using an OHS 200 Advance overhead stirrer (Velp Scientifica, Usmate Velate, Italy) until complete dissolution.

A series of eutectogels with varying concentrations of HA, XTG, and water were prepared according to the compositions outlined in Table 7. The drug concentration in the final eutectogel was set to 2.5%, *w*/*w*.

Briefly, XTG and HA were dispersed into the drug-containing NADES. The polymers were added slowly and gradually to prevent clumping, maintaining continuous stirring. Ultrapure water was gradually added, and the mixture was maintained at 75 °C for an additional 2 h under continuous stirring to ensure complete hydration and homogeneous polymer distribution. Finally, the mixture was cooled to room temperature (25 °C) to induce gelation. The resulting eutectogels were stored at room temperature for further characterization.

**Figure 18:**
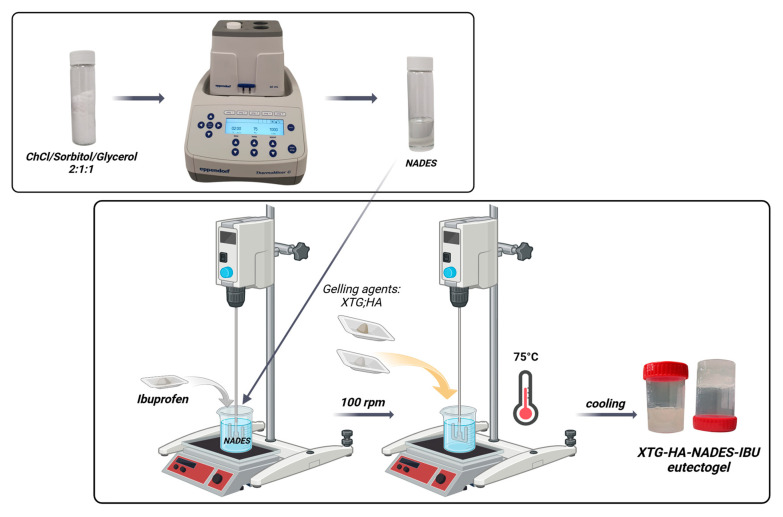
Schematic representation of the preparation process for eutectogels Created with BioRender. Anuta, V. (2025) https://BioRender.com/u83m072.

Figure 18 illustrates the step-by-step process for synthesizing eutectogels, starting from the preparation of the NADES solution to the final formation of the XTG-HA-NADES-IBU eutectogel.

### 4.3. Determination of IBU Concentration

The quantitative determination of IBU was performed using a validated HPLC method, as previously described [7]. Briefly, a Jasco 4000 Series RP-HPLC (JASCO Corporation, Tokyo, Japan) system was employed. Chromatographic separation was performed using a Kinetex^®^ C18 column (100 × 3 mm, 2.6 µm particle size; Phenomenex, Torrance, CA, USA) maintained at a constant temperature of 45 °C. An isocratic elution mode was employed, with a mobile phase consisting of 0.1% formic acid in water (mobile phase A) and a 1:1 (*v*/*v*) mixture of acetonitrile and methanol (mobile phase B). A constant mobile phase composition of 45:55 (A:B, *v*/*v*) was maintained throughout the analysis at a flow rate of 0.7 mL/min. Detection was carried out at 222 nm. The HPLC method was validated according to the current ICH guidelines [174].

### 4.4. Rheological Characterization

#### 4.4.1. Flow Behavior Analysis

The viscosity and flow behavior of the experimental systems were characterized at 37 °C using an RM100 CP2000 Plus cone-plate rheometer (Lamy Rheology Instruments, Champagne au Mont d’Or, France) equipped with a CP6020 measuring cone (Ø 60 mm, angle 2°) [115]. Flow curves were generated by subjecting the samples to a controlled increase in shear rate from 1 to 100 s^−1^. The rheological parameters, including shear rate (s^−1^), shear stress (Pa), and viscosity (Pa·s), were recorded and analyzed using the power law model (Equation (1)):

(1) $$\eta =K\cdot {\dot{\gamma }}^{n-1}$$

where *η* (Pa·s) represents the apparent viscosity, $\dot{\gamma }$ is the shear rate (s^−1^), *K* is the consistency index (Pa·s^n^), and *n* is the flow behavior index (dimensionless). The consistency index, *K*, reflects the viscosity of the fluid at a shear rate of 1 s^−1^.

The flow behavior index, *n*, provides insights into the fluid’s response to shear. A value of *n* = 1 indicates Newtonian behavior, where the viscosity remains constant regardless of shear rate. Values of *n* less than 1 (*n* < 1) are characteristic of shear-thinning or pseudoplastic fluids, where the viscosity decreases as the shear rate increases. The degree of shear-thinning behavior becomes more pronounced as the value of *n* deviates further from unity [175].

#### 4.4.2. Thixotropy Evaluation

The thixotropic behavior of the NADES eutectogels was evaluated at 37 °C using a hysteresis loop method. A shear rate ramp was applied to the samples, increasing from 1 s^−1^ to 100 s^−1^ (up curve) and then decreasing back to 1 s^−1^ (down curve). Shear stress and apparent viscosity were recorded every 5 s during both the upward and downward ramps, allowing for the construction of hysteresis loops. The area enclosed within the hysteresis loop was quantified and used as an indicator of the degree of thixotropy exhibited by the eutectogels [69,176]. A larger hysteresis loop area corresponds to a greater degree of thixotropy, reflecting a more significant breakdown and slower recovery of the gel structure under shear.

(2) $$S_{thix}=\int_{{\dot{\gamma }}_{min}}^{{\dot{\gamma }}_{max}}(\tau_{up}-\tau_{down})d\;\dot{\gamma }$$

where $\tau_{up}$ is the shear stress during the upward ramp, $\tau_{down}$ is the shear stress during the downward ramp, and ${\dot{\gamma }}_{min}$ and ${\dot{\gamma }}_{max}$ are the minimum and maximum shear rates, respectively.

In addition to *S_thix_*, the thixotropy index (*TI*, %) was introduced to provide a normalized measure of the thixotropic behavior. The thixotropy index was calculated as the ratio of the hysteresis loop area to the area under the upward curve:

(3) $$TI\;\left(\%\right)=\frac{S_{thix}}{\int_{{\dot{\gamma }}_{min}}^{{\dot{\gamma }}_{max}}\tau_{up}d\;\dot{\gamma }}\cdot 100$$

where $\int_{{\dot{\gamma }}_{min}}^{{\dot{\gamma }}_{max}}\tau_{up}d\;\dot{\gamma }$ is the area under the upward shear stress curve.

### 4.5. Texture Analysis

The textural properties of the eutectogels were evaluated using a TX-700 texture analyzer (Lamy Rheology Instruments, Champagne au Cosmetics 2024, Mont d’Or, France) equipped with a 5 kg load cell and a stainless-steel cylindrical probe (25 mm diameter, 40 mm height), as previously described [89]. Prior to analysis, approximately 30 g of each eutectogel formulation was placed in a suitable container and allowed to equilibrate to room temperature.

The textural analysis involved two consecutive compression–decompression cycles. The probe was lowered into the sample at a constant speed of 1 mm/s until a 10 mm penetration depth was achieved. The onset of sample compression was defined as the point at which the measured force exceeded 0.01 N. Following compression, the probe was raised at the same speed to a return position 25 mm above the sample surface.

RheoTex software (version 1.52.0.0) was employed to acquire and analyze the force–time data generated during the probe’s movement. The following textural parameters were calculated from the force–time curves: (i) hardness (N), defined as the peak force required to compress the sample during the first compression cycle; (ii) adhesiveness (N·s), defined as the negative area under the force–time curve during the initial probe withdrawal, representing the work required to overcome the adhesive forces between the probe surface and the sample; (iii) cohesiveness, calculated as the ratio of the positive area under the force–time curve during the second compression to that of the first compression, reflecting the internal strength of the sample; (iv) resilience, calculated as the ratio of the area under the force–time curve during the decompression phase to the area under the curve during the compression phase, reflecting the sample’s ability to recover its original shape after deformation; (v) springiness, defined as the ratio of the distance the sample recovers between the end of the first compression and the start of the second compression to the distance it was compressed during the first compression, refers to the ability of a gel to return to its original shape after a deforming force is removed; and (vi) stringiness (mm), the distance over which the gel can stretch before the sample detaches from the probe during withdrawal [89,177].

### 4.6. In Vitro Release Experiments

A flow-through cell assembly, employing a Sotax CE7 smart USP Apparatus 4 with a CP7 piston pump (Sotax AG, Aesch, Switzerland), was used to evaluate the drug release characteristics [176]. Each flow cell (22.6 mm diameter) was fitted with a 5 mm ruby bead at the cone’s apex to protect the inlet tube. To ensure uniform media flow and consistent sample contact, approximately 8.0 g of 1 mm glass beads was added to the cone area.

The experimental formulations were loaded into 0.4 mL semisolid adaptors (0.4 mL sample volume, 1.54 cm^2^ exposure area) and were fitted with a 0.45 µm pore size Supor^®^ polyethersulfone (PES) membrane disc filter (Pall Life Sciences, Portsmouth, UK). Prior to each experiment, the membranes were hydrated by soaking in the receptor medium for a minimum of 30 min. The adapters with the membrane facing down were loaded into flow-through cells. The experiments were performed using a closed-loop configuration, using 250 mL of 20% ethanol in pH 6.8 phosphate buffer (*v*/*v*) at a constant flow rate of 8 mL/min. The ethanol content was determined based on preliminary solubility tests to maintain sink conditions throughout the experiment. At predetermined time points, 1 mL aliquots were withdrawn from the circulating media and immediately replaced with an equivalent volume of fresh, preheated medium. The collected samples were subsequently analyzed using HPLC to quantify drug release.

Drug release was expressed as the cumulative amount released per unit area of the membrane over the experimental time frame.

The release kinetics were analyzed using the Higuchi model, as per the SUPAC-SS guideline [114], with the release rate determined from the slope of the linear portion of the cumulative release versus square root of time plot, corresponding to steady-state drug diffusion through the membrane. To further elucidate the drug release mechanism, the release data were fitted to multiple mathematical models, each representing distinct kinetic processes [178,179]. These included zero order, first order, Korsmeyer–Peppas, and Weibull models, each representing a distinct release pattern based on diffusion or other underlying mechanisms [116].

### 4.7. Experimental Design

A face-centered central composite design was employed to investigate the influence of three independent variables on the rheological, textural, and drug release properties of XTG-HA eutectogels. This specific design was chosen as it allows for the efficient exploration of a wide range of factor combinations and provides information about potential quadratic effects, which is important for optimizing formulation properties. The independent variables, outlined in Table 7, were the following: percentage of water added in formulation (% water—*X*_1_), percentage of hyaluronic acid (% HA—*X*_2_), and percentage of XTG (% XTG—*X*_3_).

The design included 8 factorial points, 6 axial points (*α* = 1), and 3 central replicates, totaling 17 runs. The inclusion of central replicates allowed for the estimation of experimental error and validation of model reproducibility, ensuring robust statistical analysis. The experimental design, comprising 17 analytical runs, was generated and analyzed using Design Expert version 13 software (Stat-Ease Inc., Minneapolis, MN, USA). The design matrix was executed with a randomized run order to minimize potential bias (Table 8).

The investigated response variables, representing the rheological, textural, and drug release characteristics of the eutectogels, included the following: rheological properties (consistency index (*K*)—*Y*_1_, flow behavior index (*n*)—*Y*_2_, hysteresis loop area—*Y*_3_, thixotropy index—*Y*_4_); textural properties (hardness—*Y*_5_, adhesiveness—*Y*_6_, cohesiveness—*Y*_7_, resilience—*Y*_8_, springiness—*Y*_9_, and stringiness—*Y*_10_); and drug release properties (release rate—*Y*_11_ and cumulative release at 2 h—*Y*_12_).

Multiple regression analysis, utilizing a second-order polynomial equation, was employed to establish the relationships between the independent variables and the measured responses. The general form of the polynomial equation was as follows:

$$Y_{i}=b_{0}+\sum_{i=0}^{n}b_{i}X_{i}+\sum_{i=0}^{n}b_{ii}X_{i}^{2}+\sum_{i<j}^{n}b_{ij}X_{i}X_{j}+\varepsilon$$

where *Y_i_* is the measured response; *b*_0_ represents the intercept, which is the predicted value of *Y_i_* when all the independent variables are zero; *b_i_*, *b_ii_*, and *b_ij_* are the linear, quadratic, and interaction coefficients calculated by multiple regression analysis; *X_i_* and *X_j_* represent the independent variables; and *ε* represents the random error.

The significance of the regression models and the individual model terms was evaluated using analysis of variance with a significance level of *p* < 0.05. Non-significant terms were iteratively removed from the models using backward elimination based on the Akaike information criterion [176]. The coefficient of determination (*R*^2^) was used to assess the goodness of fit for the final reduced models.

Response surface methodology, employing contour plots, surface response plots, and interaction plots, was utilized to visually represent and interpret the relationships captured by the polynomial models. This graphical analysis facilitated the identification of optimal formulation conditions for desired eutectogel properties.

### 4.8. SEM Analysis

The optimized eutectogels were examined using a TM4000Plus scanning electron microscope (Hitachi, Tokyo, Japan), operating at a voltage of 15 kV with backscattered electron detection (BSE) mode. For SEM preparation, the eutectogels were suspended in anhydrous ethanol and allowed to incubate for 5 days. The ethanol was refreshed every 24 h to ensure complete replacement of the NADES. Following this treatment, the samples were frozen and subsequently subjected to freeze drying for 24 h. The resulting xerogels were mounted onto conductive carbon tape (TED PELLA Inc., Redding, CA, USA), securely attached to aluminum SEM stubs, and analyzed without any further treatment.

### 4.9. DSC Measurements

Calorimetric experiments were performed using a DSC Q2000 system equipped with an RCS 90 cooling system (TA Instruments, New Castle, DE, USA). Measurements were performed under dry high-purity helium at a flow rate of 25 mL/min. Roughly 10 mg of the samples was analyzed, in hermetically sealed Tzero aluminum pans. A heat–cool–heat (HCH) approach at a heating rate of 10 °C/min was used in a single run, with two cycles of the samples being cooled to –80 °C and heated to 70–120 °C (depending on the analyzed sample), with 3 min of equilibration time between cycles.

### 4.10. Swelling Behavior

The swelling behavior of the experimental eutectogels was determined gravimetrically. Briefly, the samples were molded into discs of about 1.5 cm diameter and 2 mm thickness, using vertical diffusion cell spacer discs (Hanson Research, Chatsworth, CA, USA). Each disc was placed on a Whatman GF/A glass microfiber filter with a 1.6 μm pore size (Cytiva, Marlborough, MA, USA) placed inside a cell strainer with a 40 μm pore size, adapted for 50 mL Falcon tubes (Corning Inc., Corning, NY, USA). The assembly was soaked in an excess of simulated saliva (pH 6.8) [180] and maintained at 37 °C during the experiment. At predetermined intervals (0.5, 1, 2, 3, 4, 6, 8, 12, 24, and 48 h), the excess of simulated saliva was removed by centrifuging the filters at 1500 rpm for 5 min using a SL1R plus Centrifuge (Fisher Scientific, Waltham, MA, USA), and the weight of the samples was detected by an XA 210.5Y.A analytical balance (RADWAG Balances & Scales, Radom, Poland). The swelling index [181] was calculated using the following formula:

$$Swelling\;index\;(\%)=\frac{W_{s}-W_{0}}{W_{0}}\times 100$$

Where *W_s_* is the weight of the swollen gel and *W*_0_ is the weight of the initial gel sample.

### 4.11. Mucosal Ex Vivo Residence Time

The mucosal residence time of eutectogels was assessed using a modified flow–wash assay, as described by Baus et al. [142]. Freshly excised porcine buccal mucosa was secured onto a plexiglass plate using cyanoacrylate adhesives and double-sided adhesive tape and then mounted at a 90° angle. Each gel formulation (~100 μL) was precisely applied to the center of the mucosal tissue. Simulated saliva (pH 6.8) was continuously perfused over the mucosa at a physiological flow rate of 1.5 mL/min using an Ismatec IP 78010-29 digital peristaltic pump (Masterflex LLC, Radnor, PA, USA). The mucosal residence time was defined as the duration until complete detachment or disintegration of the eutectogel. The experiment was performed in triplicate.

### 4.12. Antimicrobial Activity

#### 4.12.1. Microbial Strains

Antimicrobial activity was tested on Gram-positive (*Bacillus cereus* and *Enterococcus faecium*) and Gram-negative (*Klebsiella pneumoniae*) bacterial strains isolated from the oral cavity. The clinical strains were obtained from the Microbial Strain Collection of the Faculty of Biology, University of Bucharest, Romania, and confirmed via MALDI-TOF.

#### 4.12.2. Quantitative Antimicrobial Activity

Quantitative analysis was performed by using a binary serial microdilution method in liquid medium (Tryptone soy broth for bacteria) in a 96-well plate. The concentration range was from 50 to 1.56 mg/mL. Each well was inoculated with 10 μL of microbial suspension, adjusted to 1.5 × 106 CFU/mL from 18 to 24 h grown cultures. The MIC values were established both macroscopically, as the last concentration at which no microbial growth was observed, and spectrophotometrically. The absorbance of the microbial cultures was measured at 620 nm by using a FlexStation 3 UV-Vis (Molecular Devices, San Jose, CA, USA) Spectrophotometer.

To determine the minimum bactericidal concentrations (MBC), 5 µL was spotted from the wells where no microbial growth was observed in plates with specific solid media: Aloa for *Listeria* sp., Muller Hilton for the rest of the bacterial strains, and Sabouraud for yeasts.

#### 4.12.3. Evaluation of the Gels’ Antimicrobial Properties

The antimicrobial activity was assessed according to European Pharmacopoeia 7.0, 5.1.3. Efficacy Of Antimicrobial Preservation, using the plate count method. One gram of each sample was weighed and sterilized with UV radiation for 30 min. After sterilization, the samples were inoculated with 10^5^ CFU/g. The inoculated samples were incubated for 15 min (T0) and 24 h (T24) at 37 °C, after which 1 mL of peptone water was added, the samples were vortexed, and decimal dilutions were made that were subsequently seeded in solid medium plates (plate count agar). The plates were incubated for 24 h at 37 °C. The logarithmic reduction was calculated with the following formula:

$$Recovery\;rate\;(\%\frac{CFU}{mL})=\frac{\mathrm{lg}A}{lgB}\times 100$$

where *A* is the CFU/mL for the gel sample and *B* is the CFU/mL for the positive control (strain without gel).

### 4.13. In Vivo Evaluation of Anti-Inflammatory Activity

#### 4.13.1. Animals and Housing

This study utilized 64 male Wistar rats (201 ± 25 g body weight) obtained from an accredited rodent farm (National Institute for Medical-Military Research and Development “Cantacuzino”, Bucharest, Romania). Animals were housed in plastic cages (1354G EUROSTANDARD type IV) under controlled environmental conditions (20–22 °C, 35–45% relative humidity) with ad libitum access to standard granulated food and water. Food was withheld for 2 h prior to experimental procedures.

#### 4.13.2. Ethics

This study was conducted in accordance with ethical guidelines for the use of laboratory animals in scientific research and was approved by the Scientific Research Ethics Committee of the University of Medicine and Pharmacy “Carol Davila” from Bucharest, Romania.

#### 4.13.3. Treatment Groups

The anti-inflammatory efficacy of topically administered IBU formulations was evaluated using a rat model of plantar edema. Two distinct inflammatory stimuli were employed, namely, λ-carrageenan (car) and kaolin (cao), which trigger inflammatory responses primarily mediated by pro-inflammatory prostaglandins/histamine/serotonin and pro-inflammatory cytokines (IL-1β, IL-6, TNFα), respectively [172,182].

Rats were randomly assigned to 8 groups (*n* = 8 per group). Inflammation was induced by intraplantar injection of either 1% λ-carrageenan (car) or 10% kaolin (cao) after treatment administration. Oral distilled water was given as the negative control, and commercial ibuprofen gel (containing 5% IBU) served as a positive control for topical anti-inflammatory efficacy.

The experimental groups, the inflammatory stimuli applied (carrageenan or kaolin), the treatments administered, and the corresponding routes of administration are detailed in Table 9.

#### 4.13.4. Anesthesia and Inflammation Induction

Prior to inflammation induction, rats were anesthetized with urethane (130 mg/kg body weight, intraperitoneal injection of a 13% solution). Baseline plantar volume of the right hind paw was measured, followed by the administration of the assigned treatments. Inflammation was induced by intraplantar injection of either 0.2 mL of 1% λ-carrageenan aqueous suspension (car groups) or 0.2 mL of 10% kaolin aqueous suspension (cao groups).

#### 4.13.5. Plethysmometric Measurement of Edema

Plantar edema volume was measured at 1, 2, 3, 5, and 24 h post-inflammation induction using a 76-0220 digital plethysmometer (Harvard Apparatus, Holliston, Massachusetts, MA, USA) equipped with a 3 mL cell.

Edema volume data are expressed as the mean ± standard deviation. The dynamics of induced intraplantar edema were calculated using the formula:

(4) $$Ev\%=\frac{V_{t}-V_{0}}{V_{0}}\cdot 100$$

where *V*_0_ represents the initial paw volume and *V_t_* is the paw volume at each experimental time point (t = 1, 2, 3, 5, 24 h).

### 4.14. Statistical Analysis

Data obtained in the experiments are expressed as means ± SDs. Statistical analysis was performed using GraphPad Prism version 10.0.0 for Windows (GraphPad Software, La Jolla, CA, USA). One-way ANOVA followed by Tukey’s HSD post hoc test was employed for multiple group comparisons, while Student’s *t*-test was used for comparing two groups. A *p*-value of less than 0.05 was considered statistically significant.

## Figures and Tables

**Table 1:** Rheological, textural, and kinetic properties of various XTG-HA eutectogel formulations (G1–G17) obtained by applying a face-centered central composite design, with each column representing a specific parameter: *Y*_1_ (consistency index, *K*, Pa·sⁿ), *Y*_2_ (flow behavior index, n), *Y*_3_ (hysteresis loop area, Pa·s⁻^1^), *Y*_4_ (thixotropy index, %), *Y*_5_ (hardness, N), *Y*_6_ (adhesiveness, N·s), *Y*_7_ (cohesiveness), *Y*_8_ (resilience), *Y*_9_ (springiness), *Y*_10_ (stringiness, mm), *Y*_11_ (drug release rate, μg/cm^2^/min^1/2^), and *Y*_12_ (cumulative drug release at 2 h, μg/cm^2^).

Code	*Y*_1_ (Pa·s^n^)	*Y*_2_	*Y*_3_ (Pa·s^−1^)	*Y*_4_ (%)	*Y*_5_ (N)	*Y*_6_ (N·s)	*Y*_7_	*Y*_8_	*Y*_9_	*Y*_10_ (mm)	*Y*_11_ (μg/cm^2^/min^1/2^)	*Y*_12_ (μg/cm^2^)
G1	84.6 ± 1.5	0.219 ± 0.014	1835 ± 51	10.73 ± 0.40	0.991 ± 0.021	2.36 ± 0.04	0.957 ± 0.026	0.173 ± 0.005	0.973 ± 0.036	20.79 ± 1.15	207.1 ± 3.8	1302 ± 48
G2	104.4 ± 4.8	0.203 ± 0.007	2408 ± 200	10.62 ± 0.69	1.213 ± 0.083	2.67 ± 0.20	0.921 ± 0.076	0.233 ± 0.022	0.978 ± 0.045	22.60 ± 1.04	149.1 ± 13.8	1157 ± 43
G3	25.8 ± 2.4	0.265 ± 0.015	687 ± 51	9.07 ± 0.67	0.417 ± 0.011	0.89 ± 0.07	1.007 ± 0.102	0.189 ± 0.009	0.969 ± 0.081	17.17 ± 0.95	244.2 ± 22.5	1403 ± 52
G4	108.3 ± 10	0.218 ± 0.010	2770 ± 153	11.28 ± 0.31	1.107 ± 0.089	2.98 ± 0.14	0.995 ± 0.055	0.181 ± 0.017	0.955 ± 0.044	22.60 ± 1.67	121.8 ± 2.2	1116 ± 103
G5	61.1 ± 5.1	0.231 ± 0.015	1351 ± 50	8.87 ± 0.57	0.752 ± 0.036	1.90 ± 0.04	0.971 ± 0.081	0.161 ± 0.012	0.954 ± 0.062	18.67 ± 0.52	196.3 ± 5.4	1379 ± 64
G6	131.8 ± 6.1	0.189 ± 0.017	1441 ± 53	5.76 ± 0.37	1.144 ± 0.142	2.96 ± 0.19	0.974 ± 0.036	0.209 ± 0.004	0.974 ± 0.081	18.53 ± 1.54	128.5 ± 11.9	1126 ± 104
G7	85.2 ± 3.1	0.259 ± 0.012	3230 ± 209	13.20 ± 0.85	1.174 ± 0.042	3.13 ± 0.09	0.867 ± 0.080	0.153 ± 0.007	0.986 ± 0.073	24.41 ± 0.68	197.3 ± 5.5	1232 ± 57
G8	37.2 ± 1.0	0.317 ± 0.026	1810 ± 167	13.27 ± 1.22	0.689 ± 0.044	1.70 ± 0.05	0.957 ± 0.071	0.112 ± 0.007	0.973 ± 0.045	21.70 ± 1.20	256.6 ± 23.7	1541 ± 100
G9	73.7 ± 6.1	0.212 ± 0.010	1024 ± 76	6.26 ± 0.17	0.772 ± 0.020	2.01 ± 0.07	0.957 ± 0.062	0.191 ± 0.005	0.974 ± 0.072	19.43 ± 1.26	184.8 ± 11.9	1197 ± 33
G10	116.9 ± 8.6	0.219 ± 0.014	1865 ± 34	8.46 ± 0.70	1.232 ± 0.060	2.92 ± 0.13	0.875 ± 0.032	0.185 ± 0.003	0.993 ± 0.055	23.05 ± 1.91	197.7 ± 18.2	1327 ± 98
G11	81.5 ± 6	0.223 ± 0.014	2080 ± 38	11.60 ± 0.21	0.983 ± 0.083	2.62 ± 0.15	0.940 ± 0.069	0.167 ± 0.017	0.977 ± 0.036	20.51 ± 1.70	207.1 ± 3.8	1351 ± 62
G12	94.1 ± 3.5	0.319 ± 0.018	3782 ± 105	13.50 ± 0.5	1.173 ± 0.059	3.49 ± 0.26	0.781 ± 0.050	0.184 ± 0.014	1.003 ± 0.037	26.21 ± 2.42	224.3 ± 18.6	1382 ± 77
G13	86.8 ± 7.2	0.186 ± 0.005	736 ± 48	4.38 ± 0.08	0.908 ± 0.090	2.06 ± 0.04	1.002 ± 0.037	0.244 ± 0.025	0.951 ± 0.053	16.72 ± 0.93	109.4 ± 6.1	984 ± 18
G14	150.4 ± 4.2	0.193 ± 0.005	5682 ± 105	15.85 ± 1.02	1.569 ± 0.084	3.77 ± 0.35	0.790 ± 0.015	0.248 ± 0.018	0.996 ± 0.092	24.86 ± 0.92	161.9 ± 3.0	1094 ± 101
G15	67.6 ± 1.9	0.249 ± 0.023	1076 ± 99	6.29 ± 0.41	0.783 ± 0.017	2.18 ± 0.14	0.957 ± 0.062	0.154 ± 0.013	0.985 ± 0.045	18.98 ± 0.53	230.0 ± 8.5	1394 ± 64
G16	89.9 ± 5.8	0.219 ± 0.004	1638 ± 45	9.80 ± 0.27	1.001 ± 0.085	2.62 ± 0.17	0.953 ± 0.044	0.166 ± 0.012	0.970 ± 0.090	19.89 ± 0.55	194.0 ± 16.1	1326 ± 24
G17	51.0 ± 4.2	0.260 ± 0.024	1849 ± 171	12.13 ± 0.56	0.724 ± 0.019	2.00 ± 0.15	0.926 ± 0.034	0.139 ± 0.003	0.986 ± 0.055	20.79 ± 1.73	239.2 ± 13.2	1490 ± 96

**Table 2:** Regression coefficients, *p*-values, and model statistics for the response surface models evaluating the effects of the independent variables (*X*_1_, *X*_2_, *X*_3_) and their interactions on the rheological, textural, and kinetic properties of XTG-HA eutectogel formulations.

Variable	Intercept	*X*_1_	*X*_2_	*X*_3_	*X*_1_ *X*_2_	*X*_1_ *X*_3_	*X*_2_ *X*_3_	$X_{1}^{2}$	$X_{2}^{2}$	$X_{3}^{2}$	Model	Lack of Fit	Adjusted *R*^2^
*Y*_1_	81.48	−8.95	24.17	30.58	-	-	-	-	6.53	-	-	-	0.9761
*p*-values	-	<0.0001	<0.0001	<0.0001	-	-	-	-	0.020	-	<0.0001	0.480	-
*Y*_2_	0.225	−0.020	-	−0.042	-	0.011	-	0.016	-	-	-	-	0.9337
*p*-values	-	<0.0001	-	<0.0001	-	0.013	-	0.008	-	-	<0.0001	0.091	-
*Y*_3_	1860.43	−1231.00	649.20	383.30	−473.75	−305.75				363.67	-	-	0.9303
*p*-values	-	<0.0001	<0.0001	0.004	0.002	0.026	-	-	-	0.049	<0.0001	0.314	-
*Y*_4_	10.29	−3.53	0.30	−0.64	−0.78	−0.70	1.06	-	−1.55	1.17			0.9562
*p*-values	-	<0.0001	0.190	0.016	0.010	0.017	0.002	-	0.004	0.015	<0.0001	0.848	-
*Y*_5_	0.978	−0.169	0.203	0.216	−0.043					-	-	-	0.9841
*p*-values	-	<0.0001	<0.0001	<0.0001	0.004	-	-	-	-	-	<0.0001	0.153	-
*Y*_6_	2.48	−0.50	0.58	0.42	-	-	−0.17	-	-	-	-	-	0.9589
*p*-values	-	<0.0001	<0.0001	<0.0001	-	-	0.005	-	-	-	<0.0001	0.646	-
*Y*_7_	0.931	0.051	−0.055	-	0.038	-	-	-	-	-	-	-	0.9417
*p*-values	-	<0.0001	<0.0001	-	<0.0001	-				-	<0.0001	0.228	-
*Y*_8_	0.170	0.011	0.009	0.034	−0.026	-	-	-	-	0.018	-	-	0.9447
*p*-values	-	0.002	0.005	<0.0001	<0.0001	-	-	-	-	0.001	<0.0001	0.169	-
*Y*_9_	0.976	−0.006	0.015	−0.006	−0.004	-	-	-	-	-	-	-	0.915
*p*-values	-	0.002	<0.0001	0.002	0.042	-	-	-	-	-	<0.0001	0.343	-
*Y*_10_	20.99	−2.89	1.48	-	-	-	-	-	-	-	-	-	0.9096
*p*-values	-	<0.0001	<0.0001	-	-	-	-	-	-	-	<0.0001	0.220	-
*Y*_11_	198.64	−6.50	1.41	−52.36	-	-	13.21	−12.76	-	-	-	-	0.9623
*p*-values	-	0.034	0.610	<0.0001	-	-	0.001	0.011	-	-	<0.0001	0.503	-
*Y*_12_	1323.29	−25.95	−9.97	−173.35	39.28	-	35.92	-104.10	34.50	-	-	-	0.9886
*p*-values		0.001	0.083	<0.0001	<0.0001	-	0.000	<0.0001	0.005	-	<0.0001	0.916	-

**Table 3:** Kinetic modeling parameters for drug release from XTG-HA eutectogels.

Sample	Zero Order		First Order		Higuchi		Korsmeyer–Peppas			Weibull		
	*R* ^2^	*k*_0_	*R* ^2^	*k*_1_	*R* ^2^	*k_KP_*	*n*	*R* ^2^	*k_H_*	*R* ^2^	*α*	*β*
G1	0.9265	0.067	0.9916	0.002	0.9932	2.44	0.585	0.9987	1.381	0.9906	674.3	1.007
G2	0.9224	0.049	0.9840	0.001	0.9949	1.83	0.559	0.9993	1.227	0.9962	235.7	0.774
G3	0.9310	0.070	0.9869	0.002	0.9898	2.56	0.603	0.9973	1.268	0.9862	1016.6	1.080
G4	0.8951	0.038	0.9503	0.001	0.9964	1.47	0.507	0.9961	1.410	0.9935	123.7	0.631
G5	0.9313	0.061	0.9904	0.001	0.9931	2.25	0.577	0.9993	1.339	0.9903	455.5	0.924
G6	0.9099	0.039	0.9635	0.001	0.9967	1.49	0.532	0.9984	1.205	0.9961	160.7	0.673
G7	0.9199	0.066	0.9927	0.002	0.9933	2.42	0.582	0.9972	1.393	0.9921	519.9	0.967
G8	0.9305	0.074	0.9949	0.002	0.9910	2.70	0.599	0.9978	1.369	0.9809	1189.5	1.119
G9	0.9275	0.056	0.9897	0.001	0.9937	2.06	0.579	0.9986	1.208	0.9934	334.0	0.854
G10	0.9263	0.059	0.9919	0.001	0.9934	2.19	0.578	0.9984	1.292	0.9931	394.3	0.896
G11	0.9072	0.064	0.9900	0.002	0.9964	2.42	0.551	0.9965	1.708	0.9910	329.7	0.895
G12	0.9251	0.066	0.9918	0.002	0.9907	2.47	0.584	0.9976	1.385	0.9909	640.8	1.000
G13	0.9172	0.032	0.9595	0.000	0.9979	1.24	0.536	0.9998	0.980	0.9986	180.5	0.654
G14	0.9206	0.048	0.9844	0.001	0.9904	1.81	0.577	0.9972	1.058	0.9970	260.4	0.783
G15	0.9508	0.068	0.9838	0.001	0.9845	2.43	0.638	0.9984	0.955	0.9843	1904.4	1.155
G16	0.9393	0.057	0.9934	0.001	0.9871	2.08	0.608	0.9979	1.005	0.9937	533.6	0.927
G17	0.9300	0.069	0.9943	0.002	0.9893	2.53	0.597	0.9955	1.283	0.9951	1223.2	1.100

where: *R*²—coefficient of determination, indicating the goodness of fit for each kinetic model, *k*₀—zero-order rate constant, *k*_1_—first-order rate constant, *k_KP_*—Korsmeyer–Peppas rate constant, *n*—release exponent in the Korsmeyer–Peppas model, *α*—scale parameter in the Weibull model, and *β*—shape parameter in the Weibull model.

**Table 4:** Comparison between predicted and experimental values for the rheological, textural, and kinetic responses of the optimized XTG-HA-NADES-IBU formulation. The bias percentage indicates the accuracy of the model predictions compared to the experimental results, with positive values representing overestimation and negative values representing underestimation.

	Predicted Value	Experimental Value (*n* = 6)	Bias (%)
***Y*_1_**—Consistency index (Pa·s^n^)	66.89	67.50 ± 3.86	+0.91
***Y*_2_**—Flow behavior index	0.2753	0.2607 ± 0.0106	−5.30
***Y*_3_**—Hysteresis loop area (Pa·s^−1^)	2450.3	2198.2 ± 167.6	−10.29
***Y*_4_**—Thixotropy index (%)	12.10	13.23 ± 0.72	+9.33
***Y*_5_**—Hardness (N)	0.9111	0.9779 ± 0.0823	+7.33
***Y*_6_**—Adhesiveness (N·s)	2.5663	2.2966 ± 0.1421	−10.51
***Y*_7_**—Cohesiveness	0.8864	0.8861 ± 0.0721	−0.03
***Y*_8_**—Resilience	0.1606	0.1742 ± 0.0117	+8.46
***Y*_9_**—Springiness	0.9919	0.9866 ± 0.0722	−0.53
***Y*_10_**—Stringiness (mm)	22.44	21.24 ± 1.51	−5.35
***Y*_11_**—Release rate (μg/cm^2^/min^1/2^)	245.89	237.34 ± 13.61	−3.48
***Y*_12_**—Cumulative release 2 h (μg/cm^2^)	1477.63	1608.81 ± 48.20	+8.88

**Table 5:** Rheological behavior of the analyzed systems modeled using the power law model. The table includes the consistency index (*K*), flow behavior index (*n*), and the coefficient of determination (*R*^2^) for each evaluated composition.

Sample	*K* (Pa·s^n^)	*n*	*R* ^2^
XTG-HA-NADES-IBU	67.50	0.2607	0.9989
XTG-HA-NADES	65.69	0.2407	0.9982
XTG-HA	11.82	0.3604	0.9948
NADES	0.95	0.9734	0.9959

**Table 6:** Evaluation of MICs and MBCs (expressed in mg/mL).

Sample	*B. cereus*		*E. faecium*		*K. pneumoniae*	
	MIC (mg/mL)	MBC (mg/mL)	MIC (mg/mL)	MBC (mg/mL)	MIC (mg/mL)	MBC (mg/mL)
XTG-HA	>50	>50	50	>50	>50	>50
NADES	>50	>50	50	>50	>50	>50
XTG-HA-NADES	>50	>50	50	>50	>50	>50
XTG-HA-NADES-IBU	25	>50	25	>50	>50	>50

**Table 7:** Experimental variables and responses analyzed in the central composite design.

Variable	Code	Level		
		Low (−1)	Medium (0)	High (+1)
Water (%)	*X*_1_	20	30	40
HA (%)	*X*_2_	0.25	0.5	0.75
XTG (%)	*X*_3_	1	1.5	2
Response	Code	Measuring unit		
Consistency index (*K*)	*Y*_1_	Pa·s^n^		
Flow behavior index (*n*)	*Y*_2_	-		
Hysteresis loop area (*S_thix_*)	*Y*_3_	Pa·s^−1^		
Thixotropy index (*TI*)	*Y*_4_	%		
Hardness	*Y*_5_	N		
Adhesiveness	*Y*_6_	N·s		
Cohesiveness	*Y*_7_	-		
Resilience	*Y*_8_	-		
Springiness	*Y*_9_	-		
Stringiness	*Y*_10_	mm		
Release rate	*Y*_11_	μg/cm^2^/min^1/2^		
Cumulative release at 2 h	*Y*_12_	μg/cm^2^		

**Table 8:** Experimental design matrix of the DoE approach for the study of NADES eutectogels.

Formulation Code	*X*_1_ Water (%)	*X*_2_ HA (%)	*X*_3_ XTG (%)
G1	30	0.50	1.5
G2	30	0.50	2.0
G3	40	0.25	1.0
G4	20	0.25	2.0
G5	30	0.25	1.5
G6	40	0.75	2.0
G7	20	0.50	1.5
G8	20	0.25	1.0
G9	40	0.50	1.5
G10	30	0.75	1.5
G11	30	0.50	1.5
G12	20	0.75	1.0
G13	40	0.25	2.0
G14	20	0.75	2.0
G15	40	0.75	1.0
G16	30	0.50	1.5
G17	30	0.50	1.0

**Table 9:** Study design for evaluating the anti-inflammatory efficacy of NADES-based eutectogels in a rat model of induced plantar edema.

Model	Group	Treatment	Administration
Carrageenan (car)	1-car	Water (1 mL/100 g body weight)	Oral
	2-car	Commercial 5% IBU gel (Larofen^®^)	0.5 mL, topical (right hind paw)
	3-car	XTG-HA-NADES gel	0.5 mL, topical (right hind paw)
	4-car	XTG-HA-NADES-IBU gel (2.5% IBU)	0.5 mL, topical (right hind paw)
Kaolin (cao)	1-cao	Water (1 mL/100 g body weight)	Oral
	2-cao	Commercial 5% IBU gel (Larofen^®^)	0.5 mL, topical (right hind paw)
	3-cao	XTG-HA-NADES gel	0.5 mL, topical (right hind paw)
	4-cao	XTG-HA-NADES-IBU gel (2.5% IBU)	0.5 mL, topical (right hind paw)

## Data Availability

The original contributions presented in the study are included in the article; further inquiries can be directed to the corresponding author.

## Supplementary Materials

The following supporting information can be downloaded at: https://www.mdpi.com/article/10.3390/gels11030208/s1, List of abbreviations.
