# Supplementary material for: Novel Buccal Xanthan Gum–Hyaluronic Acid Eutectogels with Dual Anti-Inflammatory and Antimicrobial Properties
Source: Gels. 2025 Mar 15;11(3):208. doi: 10.3390/gels11030208 (PMC11942315; doi:10.3390/gels11030208)
Supplement: Supplementary file 1 [file gels-11-00208-s001.zip › gels-3195999-supplementary.pdf]

List of abbreviations:

AIC – Akaike Information Criterion
ANOVA - Analysis of Variance
API – Active Pharmaceutical Ingredient
car - λ-carrageenan
cao - kaolin
CCD-FC – Face-Centered Central Composite Design
CFU - Colony-Forming Unit
ChCl – Choline Chloride
CPM - Cohesive Polydensified Matrix
DES – Deep Eutectic Solvent
DSC – Differential Scanning Calorimetry
HA – Hyaluronic Acid
HPMC – Hydroxypropyl Methylcellulose
IBU – Ibuprofen
ICH - The International Council for Harmonisation
K – Consistency Index (rheological parameter)
k_0 - Zero-Order Release Constant
k_1 - First-Order Release Constant
k_{KP} - Korsmeyer-Peppas Release Constant
K_H - Higuchi Release Constant
HPLC – High Performance Liquid Chromatography
MALDI-TOF - Matrix Assited Laser Desorbtion Ionisation – Time of Flight
MIC – Minimum Inhibitory Concentrations
MBC - Minimum Bactericidal Concentrations
NADES – Natural Deep Eutectic Solvent
n – Flow Behavior Index
PDI – Polydispersity Index
r = Pearson Correlation Coefficient
R^2 – Coefficient of Determination
SEM - Scanning Electron Microscopy
S_{thix} -Hysteresis Loop Area
SUPAC-SS - Scale-Up and Post-Approval Changes for Nonsterile Semisolid Dosage Forms
TI - Thixotropy Index
USP – United States Pharmacopeia
XTG – Xanthan Gum
α - Scale Parameter (Weibull model)
β - Shape Parameter (Weibull Model)
